# Transition Metal–(μ-Cl)–Aluminum Bonding in α-Olefin and Diene Chemistry

**DOI:** 10.3390/molecules27217164

**Published:** 2022-10-23

**Authors:** Ilya E. Nifant’ev, Ildar I. Salakhov, Pavel V. Ivchenko

**Affiliations:** 1 A.V. Topchiev Institute of Petrochemical Synthesis, Russian Academy of Sciences (RAS), 29 Leninsky Pr., 119991 Moscow, Russia; 2 Chemistry Department, M.V. Lomonosov Moscow State University, 1-3 Leninskie Gory, 119991 Moscow, Russia

**Keywords:** carboalumination, DFT, dienes, hydroalumination, heterobimetallic complexes, metallocenes, methylenealkanes, olefins, oligomerization, polymerization, polyolefins, single-site catalysts, Ziegler–Natta catalysts

## Abstract

Olefin and diene transformations, catalyzed by organoaluminum-activated metal complexes, are widely used in synthetic organic chemistry and form the basis of major petrochemical processes. However, the role of M–(μ-Cl)–Al bonding, being proven for certain >C=C< functionalization reactions, remains unclear and debated for essentially more important industrial processes such as oligomerization and polymerization of α-olefins and conjugated dienes. Numerous publications indirectly point at the significance of M–(μ-Cl)–Al bonding in Ziegler–Natta and related transformations, but only a few studies contain experimental or at least theoretical evidence of the involvement of M–(μ-Cl)–Al species into catalytic cycles. In the present review, we have compiled data on the formation of M–(μ-Cl)–Al complexes (M = Ti, Zr, V, Cr, Ni), their molecular structure, and reactivity towards olefins and dienes. The possible role of similar complexes in the functionalization, oligomerization and polymerization of α-olefins and dienes is discussed in the present review through the prism of the further development of Ziegler–Natta processes and beyond.

## 1. Introduction

In the past few decades, catalytic transformations of olefins and conjugated dienes have become mainstream processes of the petrochemical industry: polyolefins are major multi-tonnage plastics [1,2]; polydienes are still indispensable in rubber manufacturing [3,4]. Advanced technologies of polyolefins and diene rubbers are based on the coordination polymerization of α-olefins, buta-1,3-diene, and isoprene [1,2,4,5,6,7,8,9,10,11]. Selective coordination di-, tri- and tetramerization of ethylene [12,13,14,15] are also industrially important processes. Coordination oligomerization [16,17], as well as the hydro- and carboalumination [18] of higher α-olefins, are currently lab-based processes, even though they show strong industrial prospects.

Supported metal salts (primarily chlorides) or metal complexes as pre-catalysts, and organoaluminum compounds as co-catalysts, are used in the majority of these processes. After activation, the reaction mixtures contain metal alkyls and alkylaluminum chlorides. In coordination polymerization, mechanistic understanding of the reaction mechanism as a coordination/insertion of α-olefin or diene molecule at the metal–alkyl (or metal–hydride) center in stable ligand environments is generally accepted [13,16,19,20,21]. For group 4 metal polymerization catalysts, the conventional Cossee–Arlman mechanism has been expanded by including interactions between the metal center and bulky counterions (XMAO^−^, B(C_6_F_5_)_4_^−^, anionic supports, etc.) [22,23,24,25,26,27,28,29,30,31,32,33,34,35], and binuclear Zr_2_ complexes [34,36]; however, the direct participation of R_2_AlCl in the formation of catalytic species remains hypothetic, even with the results of numerous experimental and theoretical studies [37,38,39,40,41,42,43]. It is also an open question whether M–(μ-Cl)–Al species participate in the coordination polymerization of 1,3-dienes.

**Figure 1:**
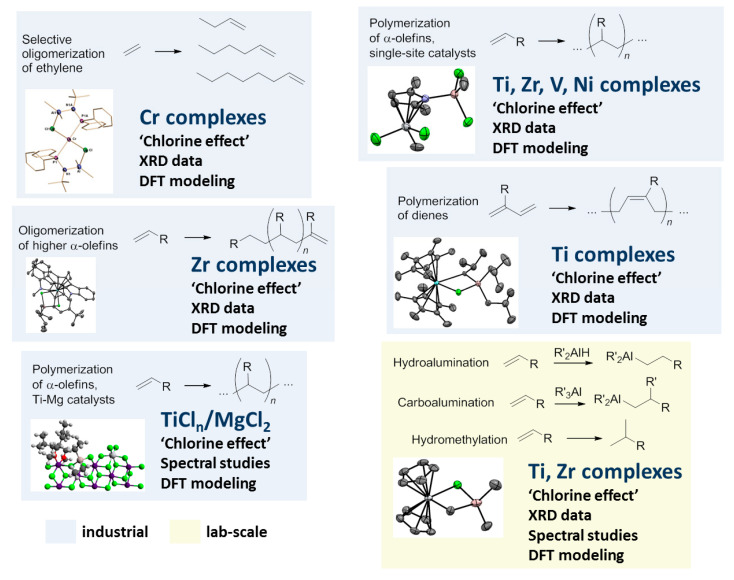
The reactions of α-olefins and dienes with the possible participation of M–(μ-Cl)–Al species.

The current view of the problem is presented in Figure 1. Despite the many reviews on α-olefin and 1,3-diene functionalization, polymerization, and oligomerization, factual information about the possible role of M–(μ-Cl)–Al bonding is either absent or presented only fragmentary. In particular, M–(μ-Cl)–Al bonding was mentioned in reviews devoted to V-catalyzed olefin polymerization [44] and the Ni-catalyzed oligomerization of olefins [45,46]. In our recent review [17], we touched upon the possible role of Zr–(μ-Cl)–Al complexes in the selective dimerization of higher α-olefins, catalyzed by (η^5^-C_5_H_5_)_2_ZrCl_2_ (Cp_2_ZrCl_2_) and *ansa*-zirconocene dichlorides in the presence of 1–10 eq. methylalumoxane (MAO). The formation of Zr–(μ-Cl)–Al species during zirconocene-catalyzed hydro- and carboalumination of α-olefins is also not in doubt; however, the specific role of M–(μ-Cl)–Al in other catalytic process remains unclear [18]. To date, the comprehensive mechanistic concept, which takes into consideration M–(μ-Cl)–Al bonding in reaction intermediates and transition states of the catalytic process, has only been developed for the Cr-catalyzed trimerization of ethylene with the use of pyrrole/Cr/Et_2_AlX (X = Cl, Et) systems [13,47,48].

In this study, we summarized disparate facts, presented in scientific periodicals, with the aim of demonstrating the fact that metal complexes, containing M–(μ-Cl)–Al fragments, are or may be directly involved in olefin and diene transformations as reagents or catalytic species. The review is divided into sections by the type of metal in the complex core.

## 2. Complexes of Ti

### 2.1. The Tebbe Reagent and Similar Ti Complexes

**Scheme 1:**
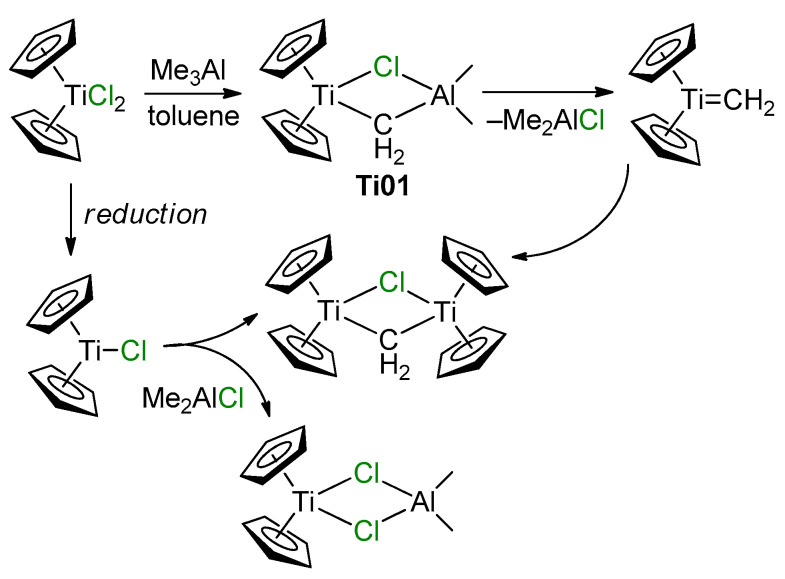
Synthesis of Tebbe reagent **Ti01** and possible side reactions and products [50].

**Figure 2:**
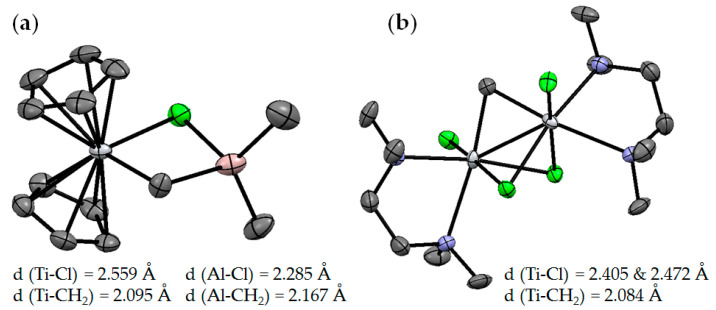
Molecular structures of (**a**) **Ti01** [50] and (**b**) **Ti02** [51]. The hydrogen atoms are omitted for clarity.

The Ti–Al heterobimetallic complex of the formula Cp_2_Ti(μ-Cl)(μ-CH_2_)AlMe_2_ (**Ti01**) was first synthesized by Tebbe et al. in 1978; the reaction of Cp_2_TiCl_2_ with AlMe_3_ resulted in the formation of **Ti01** with 80–90% yields [49]. The synthesis of **Ti01** was complicated by side reactions (Scheme 1), making it difficult to isolate the samples suitable for X-ray diffraction (XRD) analysis, and as a result, the molecular structure of **Ti01** was only determined in 2014 [50] (Figure 2a). The difference between interatomic distances (d(Ti–CH_2_) < d(Al–CH_2_), d(Ti–Cl) > d(Al–Cl)) considers **Ti01** as a complex of Cp_2_Ti=CH_2_ and Me_2_AlCl. In this way, in **Ti01,** Me_2_AlCl serves as a stabilizing agent for the Ti(IV) carbene complex, that can be removed by Lewis base.

A structural analog of the Tebbe reagent, **Ti02**, was synthesized by the reaction of the N^1^,N^1^,N^2^,N^2^-tetramethylethane-1,2-diamine (TMEDA) complex (TMEDA)TiCl_3_(THF) with [(TMEDA)ZnI]_2_CH_2_ [51]. Analysis of the molecular structure of **Ti02** (Figure 2b) showed that the value of d(Ti–CH_2_) does not differ from that in **Ti01**. Notably, a series of chlorine-free analogs of Tebbe reagent was described in [52].

**Scheme 2:**
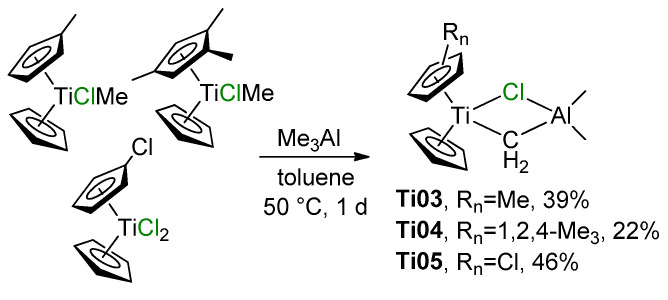
Synthesis of ring-substituted analogs of the Tebbe reagent [53].

Close structural analogs of **Ti01** were synthesized by the reactions of (η^5^-C_5_H_4_Me)CpTiClMe and (η^5^-1,2,4-C_5_H_2_Me_3_)CpTiClMe with Me_3_Al (Scheme 2, complexes **Ti03** and **Ti04**, respectively) or by the interaction of (η^5^-C_5_H_4_Cl)CpTiCl_2_ with Me_3_Al (Scheme 2, complex **Ti05**) [53]. Treatment of Cp_2_TiCl(CH=CHMe) with ^i^Bu_2_AlH resulted in the formation of complex Cp_2_Ti(μ-Cl)(μ-CHEt)Al^i^Bu_2_ [54]; the product was not isolated, but has been identified by NMR spectroscopy. The attempt of Halterman and Ramsey to synthesize the chiral Tebbe reagent from 1,1’-binaphthyl-bridged bis(η^5^-tetrahydroinden-2-yl) Ti(IV) dichloride failed [55].

**Scheme 3:**
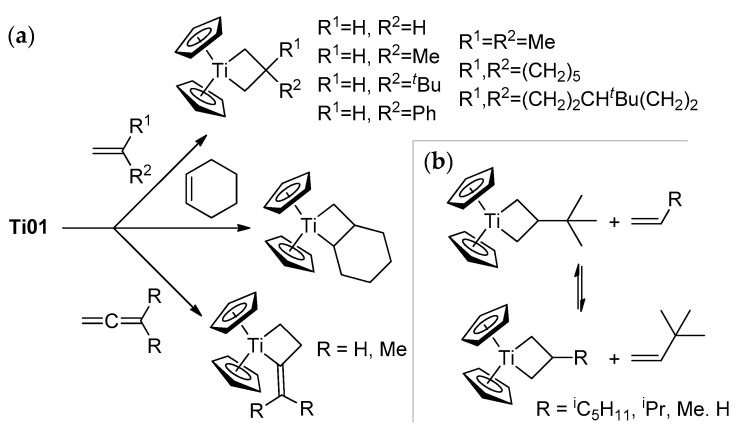
(**a**) Formation of titanacyclobutanes [59,60]; (**b**) reactivity of *tert*-butyl-substituted titanacyclobutane towards α-olefins [61].

**Ti01** has found its main applications for carbonyl methylenation similarly to phosphonium ylides in the Wittig reaction [52]. The reactions of **Ti01** with carbonyl compounds are rapid and selective, even in the presence of other reactive groups (for example, –COOR [56], –C=NOR [57]), but this subject is beyond the scope of our review. However, α-olefins can react with **Ti01**: as shown by Grubbs et al., this reaction resulted in the formation of titanacyclobutanes, and allenes formed titanacyclobutanes with exocyclic C=C bonds (Scheme 3a) [58,59,60]. Titanacyclobutanes are reactive towards α-olefins, as demonstrated by Grubbs et al.; this reversible process proceeds through the formation of Cp_2_Ti=CH_2_ [61] (Scheme 3b). Ti–Al complexes **Ti03**–**Ti05** reacted with *^t^*BuCH=CH_2_ similarly, with a formation of the mixtures of isomeric titanacyclobutanes [53].

**Scheme 4:**
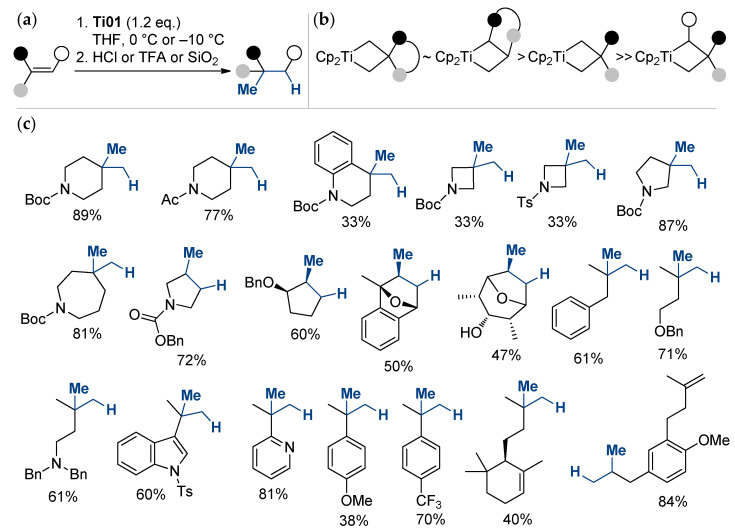
(**a**) General reaction scheme; (**b**) relative stability of titanacyclobutane intermediates; (**c**) scope and limitations of the titanium-mediated hydromethylation of alkenes [62].

A degenerate metathesis reaction between titanium methylidene unveiled from **Ti01** and unactivated alkenes, followed by acid hydrolysis, was recently proposed by Frederich et al. [62] as a direct method for the Markovnikov hydromethylation of alkenes (Scheme 4). The reaction is site-specific; the reactivity of olefins decreases in the order α-olefins > methylenealkanes > trisubstituted alkenes.

### 2.2. Single-Site Ti Catalysts of the Polymerization and Oligomerization of α-Olefins

**Scheme 5:**
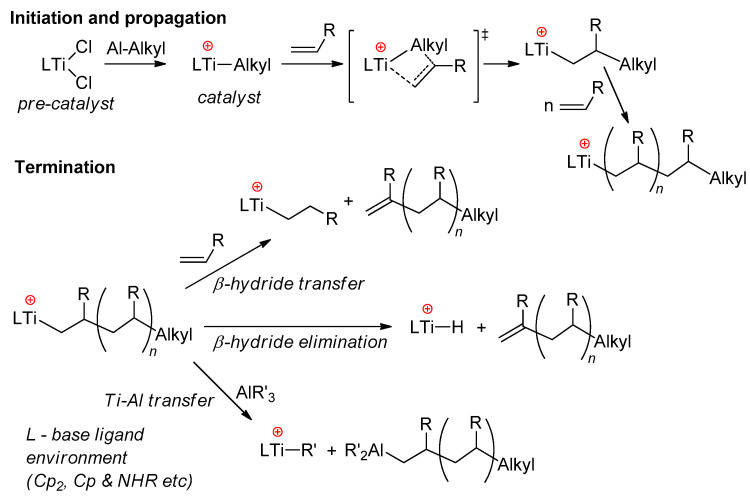
Conventional mechanism of α-olefin polymerization, catalyzed by sandwich and half-sandwich Ti(IV) complexes [65].

In early experiments on the single-site polymerization of ethylene, conducted by Shilov’s group, the occurrence of cationic species during the reaction of Cp_2_TiCl_2_ with Et_2_AlCl was regarded as an experimental criterion of the rapid catalytic process [63,64]. In the opinion of Shilov, after the intermediate formation of complex Cp_2_TiClEt·EtAlCl_2_, partial dissociation of the complex occurs, and Cp_2_TiEt^+^ is active in polymerization. For the most part, this view has not fundamentally changed in terms of the reaction mechanism, and for sandwich and half-sandwich complexes of Ti(IV), the polymerization of α-olefins includes stages of π-coordination of alkene at the Ti–Alkyl cation and insertion of the alkene via the four-membered Cossee–Arlman transition state. Possible termination events for the growing polyolefin chain are β-hydride transfer to monomer, β-hydride elimination, and Ti → Al alkyl transfer (Scheme 5) [65,66]. The main problems arising during the use of Ti(IV) sandwich complexes derive from side reactions that deactivate the active titanocene catalyst via reduction of the Ti(IV) cationic center [65]. Stabilization of the active Ti(IV) species was achieved via ligand design, with the development of half-sandwich constrained-geometry complexes (CGCs) [67,68,69,70] and different Ti(IV)-based post-metallocene catalysts [5,71].

**Scheme 6:**
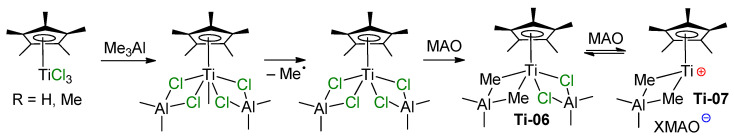
Formation of catalytically active half-sandwich Ti–Al complexes [74].

In the following model experiments on activation of Ti(IV) chloro complexes by aluminum alkyls, Ti–(μ-Cl)–Al-containing products were detected by ESR and electronic absorption spectroscopy. Thus, in the 1980s, Mach’s group studied the formation and relative stability of Ti–Al half-sandwich complexes with the formula CpTiAl_2_Cl_8–x_Et_x_ and (η^6^-C_6_H_6_)TiAl_2_Cl_8–x_Et_x_, and demonstrated their reduced stability by increasing the number of Et fragments, x [72,73]. In 2004, Bonoldi et al. studied the activation of CpTiCl_3_ and (η^5^-C_5_Me_5_)TiCl_3_ (Cp*TiCl_3_) by different aluminum alkyls, MAO and AlMe_3_/[CPh_3_][B(C_6_F_5_)_4_] [74]. ESR spectral studies have shown that during the reaction of LTiCl_3_ with Me_3_Al at an Al/Ti ratio of 10:1, after 3 h, Ti(III) complexes are formed with 12% and 8% yields, respectively. This was explained by the more electron-rich nature of Cp* in comparison with Cp, which hindered an increase in the electron density by reduction (Ti(IV) + e^−^ → Ti(III)). In the presence of MAO, complex **Ti06** was formed (Scheme 6). This complex demonstrated moderate catalytic activity in the polymerization of styrene, although it is the cationic complex **Ti07** that was considered as an *active* polymerization catalyst.

**Scheme 7:**
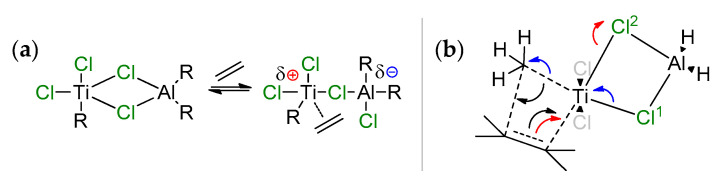
(**a**) Suggested scheme for partial ionization of the active center through action of the aluminum cocatalyst [75]; the authors proposed that after the cleavage of one of the Ti–Cl–Al bonds, the vacated position due to partial ionization is occupied through the coordination of ethylene; (**b**) ab initio modeling [78] showed that Ti–(μ-Cl)_2_–Al bonding is retained in the rate-limited transition state, and facilitates the insertion.

The hypothesis of the coordination of R_2_AlCl (Scheme 7a) with the possibility of the partial ionization of the active center, followed by the coordination/insertion of ethylene, was put forward by Jensen et al. in 1994 [75]. This hypothesis was based on the assumption that single-site Ziegler–Natta catalysts, obtained by the reaction of TiCl_4_ with AlR_3_, contain active Ti(IV) species. Quantum chemical optimization of the model complex MeCl_2_Ti(μ-Cl)_2_AlH_2_·CH_2_=CH_2_ at the DFTG level showed weak coordination of the ethylene molecule (d(Ti–C) > 3 Å) [76]; no stationary point due to the π-coordination of ethylene was found for this system. Similar results were obtained by the same research group during more thorough theoretical studies [77]; however, the authors have not abandoned the idea of the direct participation of Ti–(μ-Cl)–Al species in the catalytic process. Notably, one year earlier, Sakai reported the results of ab initio studies of the formation and further transformations of exactly the same molecule, MeCl_2_Ti(μ-Cl)_2_AlH_2_·CH_2_=CH_2_, in comparison with the MeTiCl_3_/CH_2_=CH_2_ system [78]. First, Sakai was able to find the local minima corresponding to π-complex MeCl_2_Ti(μ-Cl)_2_AlH_2_·CH_2_=CH_2_ (Scheme 7b). Second, even more interestingly, no stationary point was found for π-complex MeTiCl_3_∙CH_2_=CH_2_, and the activation energy barrier of ethylene insertion for the MeTiCl_3_/CH_2_=CH_2_ system was 20.5 kcal·mol^−1^ higher than that for MeCl_2_Ti(μ-Cl)_2_AlH_2_·CH_2_=CH_2_. Sakai explained it in this way: in the rate-limiting transition state of the ethylene insertion, the π-electrons in ethylene move to the Ti–C(ethylene) region to form the new Ti–C bond. The electrons, belonging to the Ti–C(methyl) bond, move to the C–C (methyl–ethylene) region to form a new C–C bond (push-pull mechanism, black arrows). In the Ti–(μ-Cl)_2_–Al complex, the Ti–Cl^2^ bond, opposite to the ethylene coordination side for the Ti center, increases in length with electron transfer to the Cl^2^ atom along the reaction pathway (red arrow in Scheme 7b). The original π-electrons move to the new Ti–C bond region (opposite to the Ti–Cl^2^ bond) easily (red arrow), this process is similar to S_N_2 reaction. At the same viewpoints, the Ti–Cl^1^ bond, opposite to Ti–C(methyl), decreases in length, and the electrons move from Cl^1^ to Ti and from Ti–C(methyl) bond to C(methyl) (blue arrows). Therefore, the Al–Cl (or Ti–Cl) bonds switching mechanism facilitates the push–pull mechanism of the insertion.

It can be assumed that similar single-site catalytic species are formed when using TiCl_4_/AlCl_3_ on a SiO_2_ support, activated by Et_2_AlCl [79,80]. This catalyst was studied in the oligomerization of dec-1-ene, which resulted in the preferential formation of trimers (by wt.%). The authors did not discuss the nature of the catalytic species (including oxidation state of Ti), confining themselves to the analysis of oligomer’s microstructure. However, studies of the impact of AlCl_3_/Ti ratio on catalytic activity and oligomer distribution have shown the presence of an optimum, suggesting Ti–(μ-Cl)–Al contacts. Note that dec-1-ene oligomers, obtained with the use of this Ti/Al catalytic system, were regioirregular and contained nonnegligible proportion of tail-to-tail and head-to-head monomer units, differing from structurally homogeneous products of zirconocene-catalyzed oligomerization (see Section 3.2). This fact, and uniform molecular weight distribution (MWD) of the oligomer mixtures, suggest this Ti/Al catalyst to be qualitatively distinct from conventional heterogeneous Ziegler–Natta catalysts (see Section 2.4).

The results of Jensen et al. [75] were augmented with the studies of Ti ether complexes by Bulychev’s group [81]. Here, it has clearly been established that in the presence of ethers, R_2_AlCl reduces Ti(IV) to Ti(III). A conventional activator of single-site polymerization catalysts, MAO, was completely inactive towards Ti(III) etherates. In contrast, when using Et_2_AlCl or 3:1 Et_2_AlCl/Bu_2_Mg mixture as activators, the ionic complex of Ti(III) [(15-Crown-5)TiCl_2_]^+^[AlCl_4_]^−^ (**Ti08**) demonstrated moderate activity in ethylene polymerization. The catalytic activity of **Ti08** decreases with the increasing Al/Ti ratio; therefore, it can be assumed that only partial alkylation of the Ti–Cl bonds occurs. However, there was no experimental proof of Ti–(μ-Cl)–Al bonding in catalytic species discussed in [81].

**Scheme 8:**
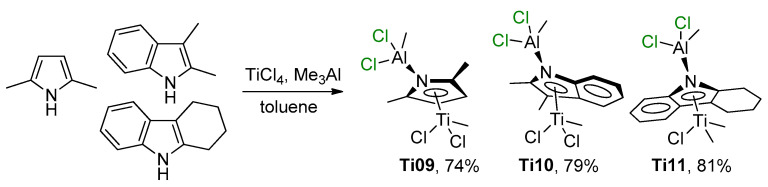
Synthesis of Ti(IV)–Al–pyrrolyl complexes **Ti09**–**Ti11** [82].

There are limited studies devoted to the participation of Ti–(μ-Cl)–Al complexes with proven structure in catalytic polymerization and oligomerization of α-olefins. In 2012, Duchateau et al. reported the synthesis, structural characterization, and ethylene polymerization performance of heterobimetallic Ti–Al–pyrrolyl complexes (η^5^-2,5-Me_2_C_4_H_2_NAlCl_2_Me)TiCl_2_Me (**Ti09**), (η^5^-2,3-Me_2_C_8_H_4_NAlCl_2_Me)TiCl_2_Me (**Ti10**), and (η^5^-3,4,5,6-C_12_H_12_NAlCl_2_Me)TiClMe_2_ (**Ti11**) [82]. These complexes were obtained by the treatment of TiCl_4_ with equimolar amounts of Me_3_Al and the corresponding pyrrole ligands (Scheme 8). 

**Figure 3:**
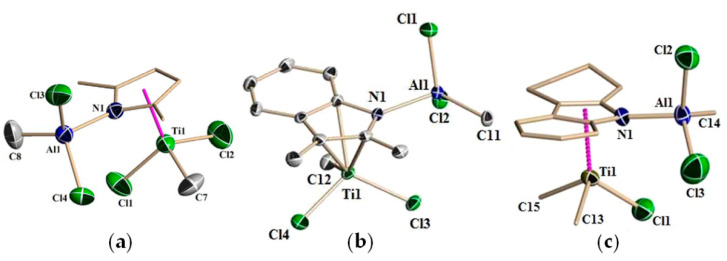
Molecular structures of Ti(IV)–Al–pyrrolyl complexes: (**a**) **Ti09**; (**b**) **Ti10**; (**c**) **Ti11**. Reprinted with permission from [82]. Copyright (2012) American Chemical Society.

Molecular structures of **Ti09**–**Ti11** were determined by XRD analysis (Figure 3). All molecules exhibited a distorted tetrahedral piano stool configuration. The Ti(1)–CEN_pyrrole_ bond distances (CEN_pyrrole_ is a centroid of the pyrrole ring) are identical in **Ti09** and **Ti10** (d(Ti(1)–CEN = 2.062 Å), but are slightly shorter than the Ti(1)–CEN_pyrrole_ bond distance in **Ti11** (d(Ti(1)–CEN = 2.088 Å). Although formally **Ti09**–**Ti11** were assigned to η^5^-type complexes, in **Ti10** and **Ti11,** titanium has a stronger interaction with the N atom, resulting in an η^4^,κ-bonding mode for Al–indolyl and Al–carbazolyl ligands. 

Upon activation with MAO at 30 °C, **Ti09** produces a single-site catalyst affording ultra-high-molecular weight polyethylene (UHMWPE, *M*_w_ = 2300 kDa, *Đ*_M_= 2.5). When **Ti09** was activated with [Ph_3_C]^+^[B(C_6_F_5_)_4_]^−^ and TIBA (50 eq.) at 30 °C, bimodal distribution of the polymer was detected, indicating the formation of two distinct active species. A similar bimodal distribution and significant reduction in *M*_n_ were observed when MAO-activated polymerization was carried out at 60 °C. MAO-activated Ti–Al complexes **Ti10** and **Ti11** displayed higher catalytic activity compared with **Ti09,** and also produced UHMWPE. Complex **Ti10** showed the same bimodal behavior when activated with [Ph_3_C]^+^[B(C_6_F_5_)_4_]^−^ and TIBA.

**Scheme 9:**
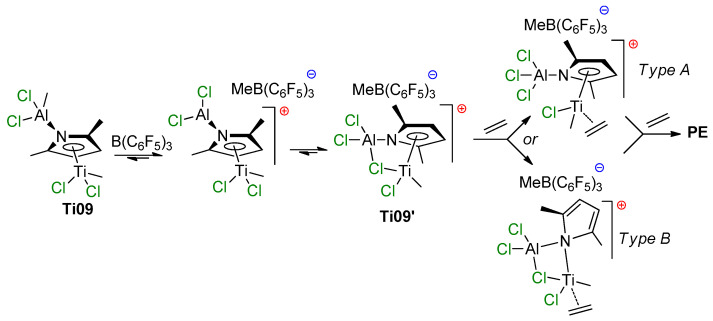
Proposed pathways of the activation of **Ti09** by B(C_6_F_5_)_3_ [82].

To clarify polymerization mechanism, the reaction of **Ti09** with B(C_6_F_5_)_3_ was investigated to determine which of the methyl groups (Ti–Me or Al–Me) would be abstracted. The ^1^H NMR spectrum clearly showed that B(C_6_F_5_)_3_ selectively abstracts Al–Me group with the formation of a *cationic* complex **Ti09′,** containing a Ti–(μ-Cl)–Al fragment (Scheme 9). The ability of the supposed cationic species **Ti09′** to polymerize ethylene was tested by treating **Ti09** with B(C_6_F_5_)_3_ under ethylene pressure (20 bar) at room temperature, and the rapid formation of polyethylene was detected.

DFT optimization of the putative structures of the cationic species and their ethylene adduct showed that a constrained type of geometry with the Ti–(μ-Cl)–Al fragment is the most stable configuration for **Ti09′**. This saturated complex **Ti09′** should not be an active catalyst; either release of the bridged Cl (Type A structure in Scheme 9) or η^5^→μ ring slippage of the pyrrole moiety (Type B structure in Scheme 9) may result in a coordinatively unsaturated complex to coordinate and polymerize ethylene. The DFT calculations showed that the formation of a Type A complex requires 12.2 kcal·mol^−1^, and no local minima were found for the Type B product of η^5^→μ ring slippage. 

**Scheme 10:**
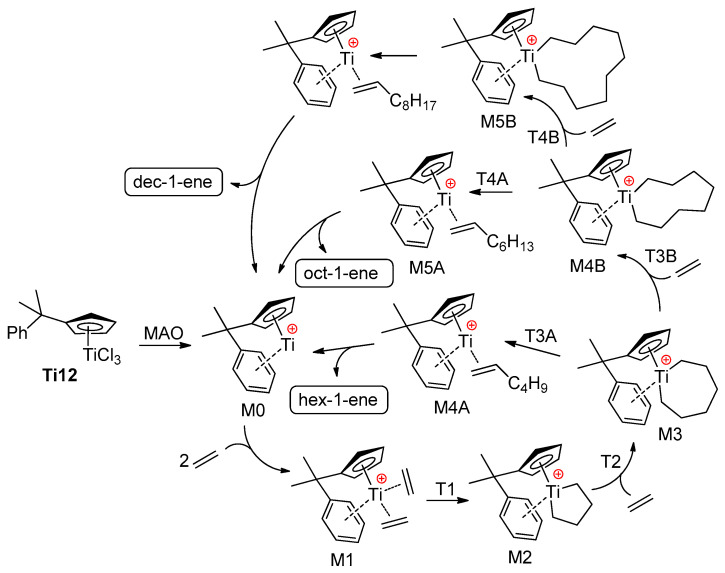
Reaction pathway for the oligomerization of ethylene, catalyzed by **Ti12**/MAO [83].

In this way, intramolecular Ti–(μ-Cl)–Al bonding has a strong impact on the catalytic behavior of Ti complex. The other extreme is weak Ti⋯Cl–Al coordination; de Bruin et al. [83] carried out a computational study of the influence of ‘chlorinated’ MAO anions on the catalytic behavior of complex **Ti12** which represents an efficient pre-catalyst of the selective trimerization of ethylene with the formation of hex-1-ene [14,15,84]. The key intermediates of the reaction are presented in Scheme 10, although the energetically unfavorable process of but-1-ene elimination from intermediate M2 (see below) is not shown.

**Figure 4:**
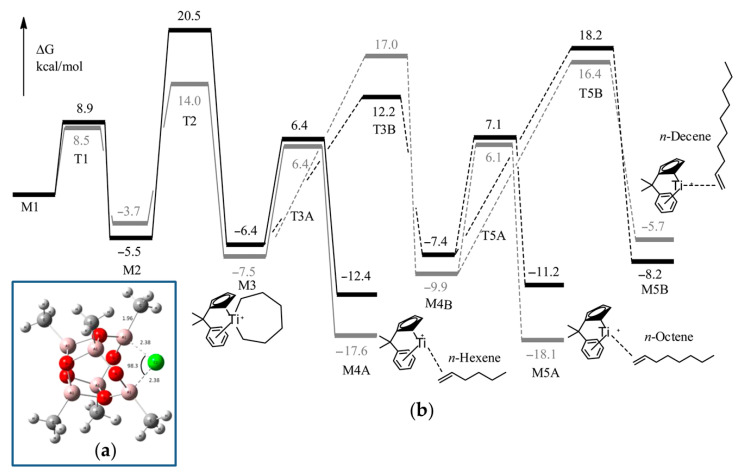
(**a**) B3LYP-optimized configuration of the [MAO-Cl]^−^ anion; (**b**) comparative reaction profile over the potential energy surface for a cationic (gray) and zwitterionic (black) catalyst. The solid line indicates the main path; the dotted line indicates the less favorable alternative path. Reprinted with permission from [83]. Copyright (2015) American Chemical Society.

For DFT calculations, one of many possible MAO structures [85,86] was selected, namely, the cage (MeAlO)_6_Cl^−^ model (Figure 4a), negatively charged due to the coordination of one Cl^−^, abstracted from **Ti12** during activation. Two different potential free energy reaction profiles have been modeled (Figure 4b): the first only takes into account the cationic catalyst (cationic profile), whereas the second describes the energy changes in the presence of a chlorinated MAO (zwitterionic profile). The reaction starts with the activated catalyst **M1** (two ethylene molecules coordinated to the cationic Ti(II) center). After oxidative coupling (**M2**), a third ethylene molecule coordinates and inserts with a formation of the seven-membered metallacycle (**M3**). This species can undergo a β-hydride transfer followed by reductive elimination (**M4A**), which, in turn, releases hex-1-ene. Alternatively, a fourth ethylene may coordinate to **M3** and insert to yield a nine-membered metallacycle **M4B**. This latter species can then undergo a ring-opening reaction to yield 1-octene (**M5A**), or a fifth ethylene coordination and insertion can take place. The calculated barrier for β-hydride elimination in **M2** (but-1-ene formation) was ~35 kcal·mol^−1^. Based on a comparison of cationic and zwitterionic profiles, it can be concluded that the presence of (MeAlO)_6_Cl^−^ does not impact the selectivity of ethylene oligomerization. Based on the results of modeling, the authors concluded that the presence of MAO model does not necessarily need to be taken into account, and the cationic system alone is sufficiently representative.

**Figure 5:**
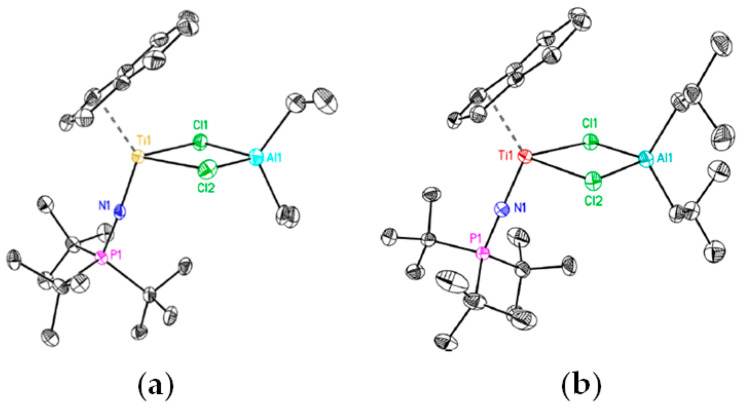
Molecular structure of Ti–Al complexes (**a**) **Ti13** and (**b**) **Ti15**. Reprinted with permission from [88]. Copyright (2021) American Chemical Society.

As reported by Blakemore et al., the reaction of the Ti(IV) complex (η^6^-C_9_H_7_)TiCl_2_(N=P*^t^*Bu_3_) with Et_3_Al resulted in the formation of Ti(III)-Al complex **Ti13** [87]. The molecular structure of **Ti13** is presented in Figure 5a. Both complexes catalyzed the polymerization of ethylene being activated by Et_3_Al in the presence of a solid superacid. During subsequent research [88], the complexes **Ti14** and **Ti15** of the formula (η^6^-C_9_H_7_)Ti (N=P*^t^*Bu_3_)(μ-Cl)_2_AlR_2_ (R = Me and ^i^Bu, respectively) were obtained, and the molecular structure of **Ti15** (Figure 5b) was virtually identical to the molecular structure of **Ti13**. However, in experiments on the polymerization of ethylene, catalytic activities of the complexes decreased in the order **Ti15** > **Ti13** > **Ti14**. In this way, the nature of the substituents at Al atoms influenced the catalyst’s properties, thus indirectly confirming the presence of Ti–(μ-Cl)–Al bonding in catalytic species.

### 2.3. Single-Site Ti Catalysts of Polymerization of Dienes

Ti(IV) halogenides, activated by aluminum alkyls, represent the first-generation catalysts of coordination polymerization of 1,3-dienes [8,89]. However, despite half a century of research, molecular structure of the catalytic species, formed as a result of the reaction of LTiCl_n_ with R_m_AlCl_3–m_, is not entirely clear. In examining the reaction mechanisms of diene polymerization, the ‘pure’ cationic concept is still actual [90]. 

**Scheme 11:**
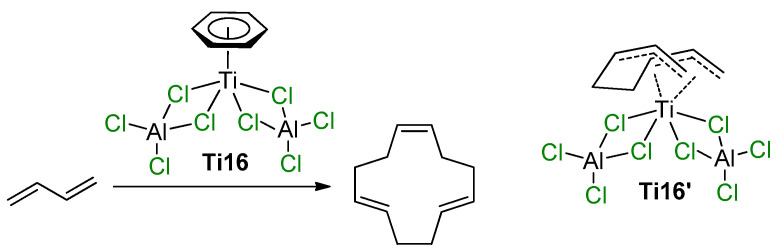
Selective cyclotrimerization of buta-1,3-diene, catalyzed by **Ti16**, and tentative structure of the intermediate **Ti16′** [91].

The participation of Ti–(μ-Cl)–Al species in the coordination polymerization of conjugated dienes was proposed by Mach et al., who studied the catalytic activity of η^6^-arene Ti complexes with AlCl_3_ and alkylaluminum chlorides [91,92,93]. Similar complexes of (η^6^-C_6_H_6_)Ti[(μ-Cl)_2_AlCl_2_]_2_ (**Ti16**) and (η^6^-C_6_Me_6_)Ti[(μ-Cl)_2_AlCl_2_]_2_ (**Ti17**) were obtained and structurally characterized in the late 1970s and early 1980s by Thewalt et al. [94,95]. The molecules of **Ti16** and **Ti17** exhibit square pyramidal geometry, with the titanium atom above the rectangle of the bridging Cl atoms and with the π-bonded arene at the apex. When **Ti16** was used as a single-component catalyst of the oligomerization of buta-1,3-diene, (1*Z*,5*E*,9*E*)-cyclododeca-1,5,9-triene was formed with 92% selectivity (Scheme 11) [91]. The authors assumed that the high stereospecificity of the cyclotrimerization indicates that the reaction proceeds by a sterically controlled coordination mechanism. The experimental results, i.e., the approximate second-order dependence of the reaction rate on buta-1,3-diene concentration, the low concentrations of all the intermediates participating in the catalytic steps and no interaction between the catalyst and final product, suggest that cyclotrimerization may proceed via the replacement of C_6_H_6_ by two molecules of buta-1,3-diene, followed by the formation of the active species **Ti16′** (Scheme 11). In the presence of EtAlCl_2_, Et_2_AlCl, and Et_3_Al, catalytic activity of complex **Ti16** increased due to the substitution of non-bridged Cl atoms by Et groups [92]. In the presence of a large excess of Et_3_Al, complete deactivation of the catalyst was observed. Complex **Ti17** demonstrated low activity due to higher stability of the (η^6^-C_6_Me_6_)–Ti bond in comparison with the (η^6^-C_6_H_6_)–Ti bond [93]. However, the substitution of non-bridged Cl atoms by Et groups proceeded stepwise with maximum preservation of the molecular symmetry—by an example of the complex (η^6^-C_6_Me_6_)Ti[(μ-Cl)_2_AlClEt]_2_ (**Ti17′**), for which XRD analysis was performed. This substitution resulted in a manyfold increase in the catalytic activity [93]. 

In conclusion, we note that Ti complexes are not actually considered as prospective catalysts of the polymerization of conjugated dienes, and further comprehensive studies of the reaction mechanism seem unlikely. 

### 2.4. Heterogeneous Ti Ziegler–Natta Catalysts

**Scheme 12:**
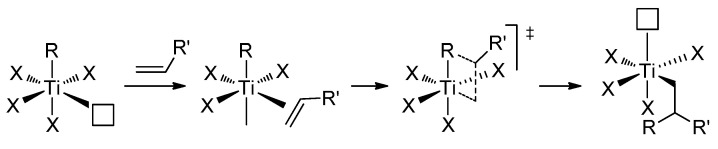
Cossee–Arlman mechanism of α-olefin polymerization [97,98]; white square indicates the vacancy of the Ti(III) atom.

The TiCl_3_/R_3_Al system, called the ‘1st generation Ziegler–Natta catalyst’, was one of the early efficient catalysts of α-olefin polymerization [96]. It is for these catalysts that the common mechanism of the coordination polymerization of α-olefins was proposed by Cossee and Arlman [97,98]. This conventional mechanism (Scheme 12) involves the participation of Ti(III)–Alkyl species and is similar to the mechanism presented in Scheme 5, except that the Ti atom has zero charge and an oxidation state of +3.

**Scheme 13:**
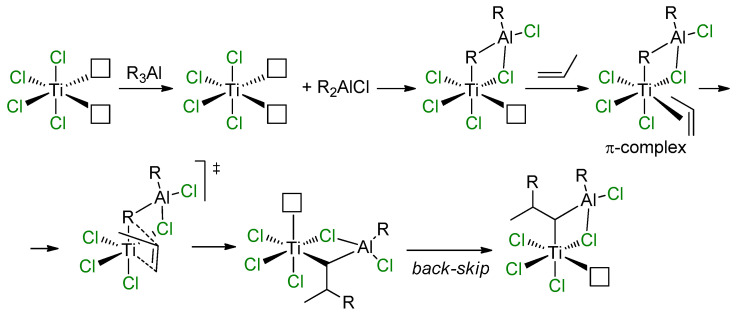
Mechanistic concept of Rodriguez and van Looy [99].

In approaching the topic of modern and efficient Ziegler–Natta (ZN) catalysts, tributes should be paid to the work of Rodriguez and van Looy, who formulated heterobimetallic concepts of the reaction mechanism to explain the impact of alkylating agents on stereospecificity of TiCl_3_ catalyst. This concept was based on an assumption of the presence of Ti centers with two potential vacancies on the Ti atom at the surface of TiCl_3_ (coordination number of the Ti atom CN_Ti_ is equal to 4) [99]. According to this concept (Scheme 13), after alkylation by R_3_Al, a Ti–(μ-Cl)–Al complex is formed (CN_Ti_ = 5). π-Coordination and subsequent insertion of the α-olefin molecule results in an alkyl complex, which further isomerizes with occupation of the position previously occupied by the R group, thus forming the isotactic polyolefin.

It has been nearly 70 years since the pioneering works of Ziegler and Natta [100,101], and there have been several generations of ZN catalysts. At present, the polymerization of α-olefins with the use of heterogeneous Ti-based MgCl_2_-supported Ziegler–Natta catalysts (TMCs) is the most important industrial chemical process in the polyolefin industry [102,103,104,105,106,107,108]. However, despite the importance and impact of TMCs, these catalyst systems have not yet been completely explored. TMCs comprise the MgCl_2_ support, the active Ti(III) [11,109,110], and Ti(II) [11,111] species; organoaluminum species formed from trialkylaluminum alkylating agent R_3_Al, and Lewis base donors. Due to the practical importance of TMCs, thus far, thousands of articles and dozens of reviews have been published; in this section, we restrict ourselves to listing several examples of the possible or proven (very rarely) participation of R_2_AlCl species in TMC-catalyzed polymerization.

**Figure 6:**
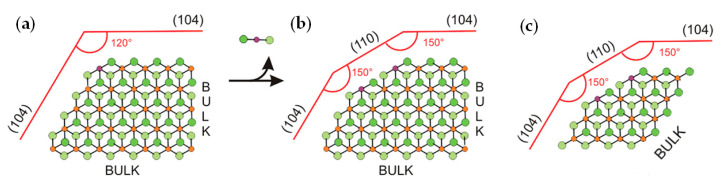
(**a**,**b**) The model for the MgCl_2_ layers developed by Cavallo et al. [110]; (**c**) the modified MgCl_2_ model with four layers instead of six employed in [102]. Reprinted with permission from [102]. Copyright (2014) Wiley-VCH Verlag GmbH & Co.

Catalytic processes occur on the TMC surface. Pre-catalysts represent TiCl_4_ adsorbed on MgCl_2_ crystallites, and the binding strength and coordination environment of the Ti atom depend on the crystal structure of the MgCl_2_ support. In their fundamental study [110], Cavallo et al. assumed the MgCl_2_ bulk to be in the α-crystalline phase with the surface comprising (104) lateral cuts (Figure 6a) that have five-coordinate magnesium centers. The removal of one MgCl_2_ unit results in a new (110) surface that can coordinate the titanium species (Figure 6b). Vanka et al. proposed the simpler model structure of the MgCl_2_ support, close in meaning to Cavallo’s model [102] (Figure 6c). In their subsequent publication, Cavallo et al. upgraded their MgCl_2_ model by (104)-facets presenting low-energy step-defects as potential sites for the strong coordination of TiCl4 [103].

After the absorption of TiCl_4_, followed by the reaction with R_3_Al, Ti(III)–Cl and Ti(III)–R species are formed [102]. Recent experimental DR UV/vis and NEXAFS data coupled with DFT simulation indicated that the majority of Ti sites in MgCl_2_/TiCl_4_ and MgCl_2_/ethyl benzoate/TiCl_4_ pre-catalysts are reduced by Et_3_Al mostly to monomeric pentacoordinated Ti(III) Cl_5_ species and, to a minor extent, to alkylated Ti(III) Cl_4_Et species. For MgCl_2_/dibutyl phthalate/TiCl_4_ pre-catalyst, Et_3_Al additionally promoted the formation of small TiCl_3_ clusters with low catalytic activity [112].

The above-mentioned articles considered two-component systems MgCl_2_/TiCl_3_, but their results are relevant to the subject of our review, determining Ti-containing species capable of Ti–(μ-Cl)–Al bonding. However, real TMCs may contain absorbed organoaluminum species. During the study of the activation of the TiCl_4_/MgCl_2_ pre-catalyst by Et_3_Cl, Oct_3_Al, and Et_2_AlCl with the use of magic-angle spinning (MAS) ^27^Al NMR [113], Potapov et al. detected three types of alkylaluminum chloride species. The changes in ^27^Al NMR MAS spectra of the TMC before and after polymerization indicated that part of the Al compounds was removed from the catalyst surface or that the coordination environments of Al in these compounds became disordered. In the authors’ view, the existence of Al compounds unremovable from the catalysts surface indicates the presence of inactive Ti–(μ-Cl)–Al species; in other words, R_2_AlCl only acts as a catalyst inhibitor.

**Figure 7:**
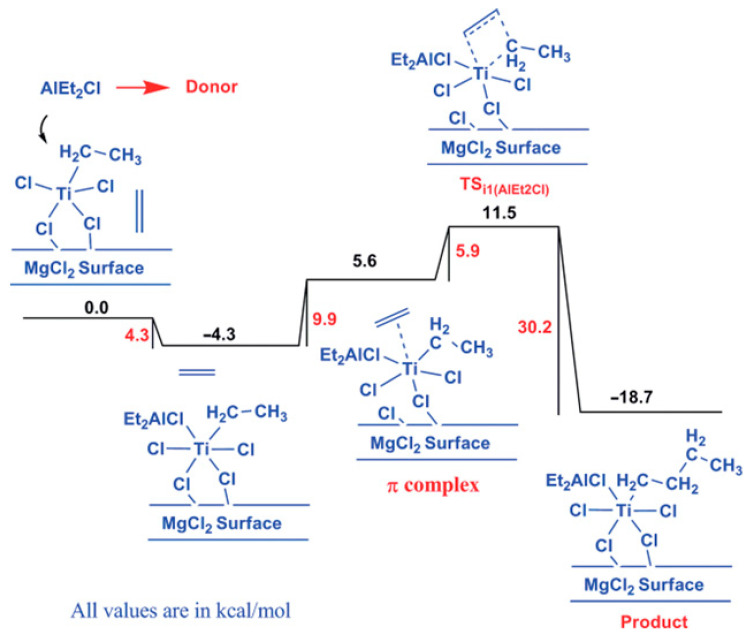
The free energy profile for the insertion of ethylene into the Ti–CH_2_CH_3_ chain with an AlClEt_2_ group coordinated to the Ti center. Reprinted with permission from [102]. Copyright (2014) American Chemical Society.

The same or a similar point of view received further development in a number of studies. In their theoretical study of the active sites in TMC [102], Vanka et al. used a simplified model of the MgCl_2_ surface (Figure 6c) and optimized the geometries of different TI(III)–Et catalytic species that are formed during the polymerization of ethylene, including the presence of the EtO^−^ ligand, ester donors, *or* Et_2_AlCl. In the absence of additives, the calculated difference in free activation energies Δ*G*^≠^ for the propagation and termination stages was only 1.2 kcal·mol^−1^. In the presence of esters, the difference increased to 12–13 kcal·mol^−1^ and was only 3 kcal·mol^−1^ when Et_2_AlCl served as a donor. In the latter case, Ti–(μ-Cl)–Al coordination resulted in a substantial increase in the Δ*G*^≠^ of ethylene insertion (15.8 kcal·mol^−1^, see Figure 7). The results of this simulation explained how polymers can be successfully produced by TMCs without alkoxy groups and donor molecules [106]. In summary, the authors suggested that the possible role of organoaluminum activators is not limited by the reduction of Ti(IV); R_2_AlCl can act as a donor due to its relatively weak Ti–(μ-Cl)–Al binding.

**Figure 8:**
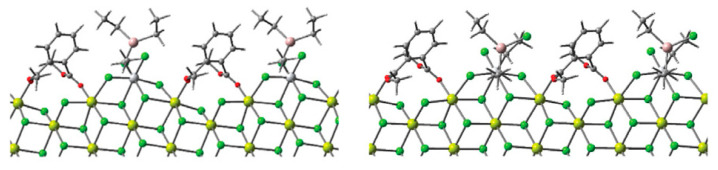
Optimized geometries of Ti–(μ-Cl)_2_–Al and Ti–(μ-Cl)(μ-Et)–Al species formed during the alkylation of Ti chloride at the (110) surface of MgCl_2_. Reprinted with permission from [114]. Copyright (2017) Elsevier B.V.

In 2017, Linnolahti et al. reported the first quantum chemical description of the initial steps of olefin polymerization on TMCs involving all the relevant catalyst components—absorbed Ti chlorides, electron donor, and R_3_Al [114]. They demonstrated that TiCl_4_ is coordinated on the (104) surface of MgCl_2_ as a binuclear Ti_2_Cl_8_ and on the (110) surface as a mononuclear TiCl_4_ (CN_Ti_ = 6 for both binding modes). Et_3_Al coordinates to the MgCl_2_ surface via an unsaturated Cl atom to initiate Ti alkylation. After alkylation via the Ti–(μ-Cl)–Al transition state on the (104) surface, Et_2_AlCl is released from the MgCl_2_ surface and dimerizes (exergonic process). However, in the presence of dimethyl phthalate as a model donor molecule at the (110) surface, the formation of Ti–(μ-Cl)_2_–Al and then Ti–(μ-Cl)(μ-Et)–Al species becomes energetically favorable (Figure 8). However, even in this case, the process is finished by Et_2_AlCl desorption. However, DFT modeling, presented in [114], closes by examining alkylation stage with a formation of Ti(IV)–Et species, the further reduction with a formation of active Ti(III) centers, as well as ability of these centers to conduct Ti–(μ-Cl)–Al bonding, remains open.

A recent study by Boisson [115] provided answers to certain questions about the role of organoaluminum activators in TMC-catalyzed polymerization. Based on the results of the study of the microstructure and MWD of polyethylenes, the authors suggested that Ti–(μ-Cl)–Al bonding may need to be considered in addressing the issue of the nature of ZN catalysts.

**Figure 9:**
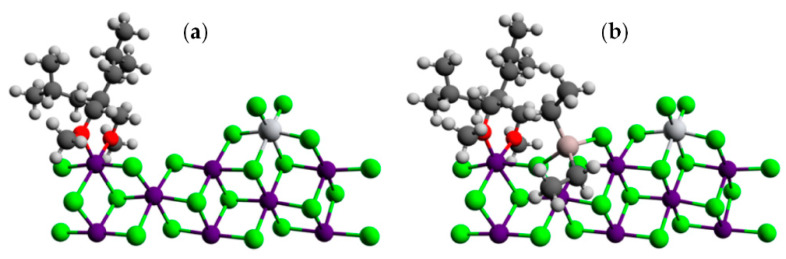
(**a**) DFT model of adjacent TiCl_4_ and 2,2-diisobutyl-1,3-dimethoxypropane co-adsorption on a MgCl_2_(110) edge; (**b**) same as panel a after the adsorption of an AlEt_2_Cl molecule. Reprinted with permission from [116]. Copyright (2017) American Chemical Society.

In [116], Busico’s group reported the results of an extensive experimental and theoretical investigation of the latest-generation commercial TMC. During this study, the researchers proposed a new function of R_2_AlCl species, namely, interactions with absorbed internal donors that provide a higher degree of stereocontrol: for the insertion of prop-1-ene at the catalytic species formed by alkylation and reduction of the TiCl_4_ precursors in panels (a) and (b) of Figure 9, the calculated ΔG_re/si_ values were ≈0 and 1.5 kcal·mol^−1^, respectively; the latter was in good agreement with the experimental data.

**Figure 10:**
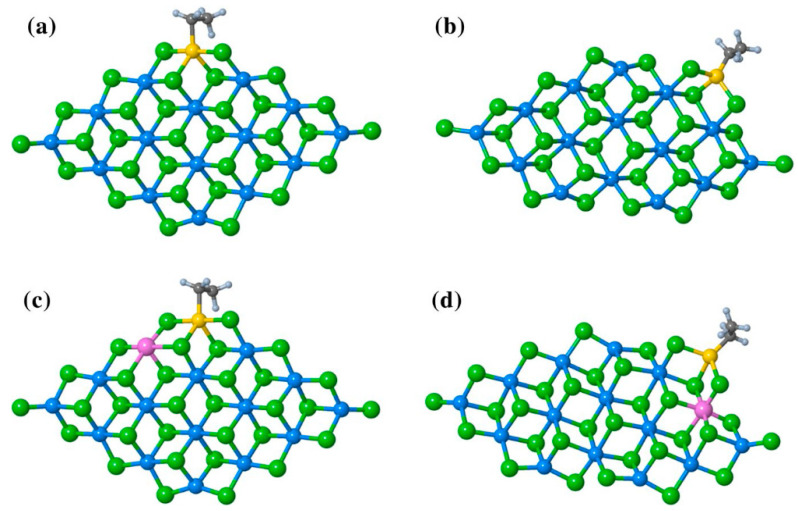
The structure of TiCl_2_Et adsorbed on (**a**) undoped (110); (**b**) undoped (104); (**c**) Al-doped (110); and (**d**) Al-doped (104) surfaces. Reprinted with permission from [117]. Copyright (2022) Springer Nature.

At the end of this section, it is appropriate to mention the most recent studies by Bahri-Laleh’s group [117,118]. In [117], ethylene coordination/insertion at different TiEt and TiEt–(μ-Cl)_2_–Al sites (Figure 10a–d) was modeled. Ethylene coordination at the Ti center on the (110) surface was thermodynamically more favored in comparison with it on the (104) surface due to the more acidic nature of 4-coordinated surface Mg atoms in (110). π-complexation of the ethylene molecule was more exergonic in Al-doped catalysts. The activation energy values for the insertion of ethylene molecule were 8.3 (a), 11.6 (b), 6.1 (c), and 9.5 (d) kcal·mol^−1^. In this way, Al-doping increased the energy barrier needed for olefin insertion. In addition, the authors proposed that absorption of the Ti chloride at the (104) surface should not be ignored when analyzing distribution of the active sites, which is in contrast to the established paradigm. However, because TMCs represent a very striking example of the ‘catalytic black box’, any substantial expansions of the existing worldview can only be welcomed.

In [118], the catalytic activity of TMCs when using different organoaluminum activators (Et_3_Al, Et_2_AlCl, EtAlCl_2_, and ^i^Bu_3_Al) was studied experimentally, and the effect of Ti–(μ-Cl)–Al bonding on catalytic activity was evaluated theoretically for Ti centers at the (110) MgCl_2_ surface and these four organoaluminum compounds. From the results of the modeling, Et_2_AlCl was the best Al component among others.

Concluding this section of the review, we have to note that, with the sole exception of complexes **Ti09**–**Ti11** [82], the question of the participation of Ti–(μ-Cl)–Al species in catalytic transformations of α-olefins still has no clear answer. Irrelevance of this question to Ti-based post-metallocenes (too high CN_Ti_) and fundamental unknowability of the TMC (the most important Ti-based catalyst) leave room for speculations, not complete answers. However, in the field of Zr-based *polymerization* catalysts we are facing a slightly better situation.

## 3. Complexes of Zr

Zirconocenes have a special place among Zr-based catalysts used in α-olefin chemistry. The hydro- and carboalumination of α-olefins [18], single-site polymerization with the use of MAO and perfluoroborate activators [119], selective dimerization [37,41,42] and oligomerization [16,17] of higher α-olefins—this is not a complete list of the processes with relevance for laboratory and industry. Historically, two groups of zirconocene-based catalytic species stand apart by the criterion of the charge of the catalytic Zr center. The first, zero-charged, group is traditionally considered as catalysts of hydroalumination, carboalumiation, and cyclocarboalumination of α-olefins and related reactions, limitedly used in the laboratory [18]. The second, ‘cationic’ species, represents highly efficient single-site α-olefin polymerization catalysts with a great potential of industrial applications [1,7,120,121]. Therefore, it is no surprise that the second group of the Zr catalysts attracted and continue to attract the most research attention; however, the participation of Zr–(μ-Cl)–Al species in catalytic process has only been proven experimentally for ‘neutral’ zirconocene species.

### 3.1. Zirconocene Chemistry: Non-Charged Complexes

The mechanisms of zirconocene-catalyzed hydroalumination and carboalumination of α-olefin were discussed ten years ago in the review by Parfenova et al. [18]. In this section, we consider only some key studies, published before 2010, and discuss the results of recent studies in more detail.

Before discussions of hydroalumination and carboalumination processes, it should be noted that the reaction of L_2_ZrCl_2_ with R_3_Al until recently was considered as the natural and only possible pathway of the activation of metallocene dichlorides. When studying this reaction, it was found that the nature of the η^5^-ligand significantly affects the completion of the process. As shown by Beck and Brintzinger [122] for alkyl exchange reactions (Equation (1)), the values of apparent equilibrium constants *K*_obs_ were 0.005 (L = η^5^-C_5_Me_5_), 0.016 (L = η^5^-C_5_H_4_*^t^*Bu), 0.19 (L = η^5^-C_5_H_4_*^t^*SiMe_3_), 0.49 (L = Cp) and 1.2 (L_2_ = *rac*-Me_2_Si(Ind)_2_). Both steric and electronic factors affect this equilibrium, and in particular, decreased electron density at the Zr atom appears to favor uptake of the Me group.

2 Cp_2_ZrCl_2_ + Al_2_Me_6_ ⇆ 2 Cp_2_ZrMeCl⋯AlMe_2_Cl (1)

**Figure 11:**
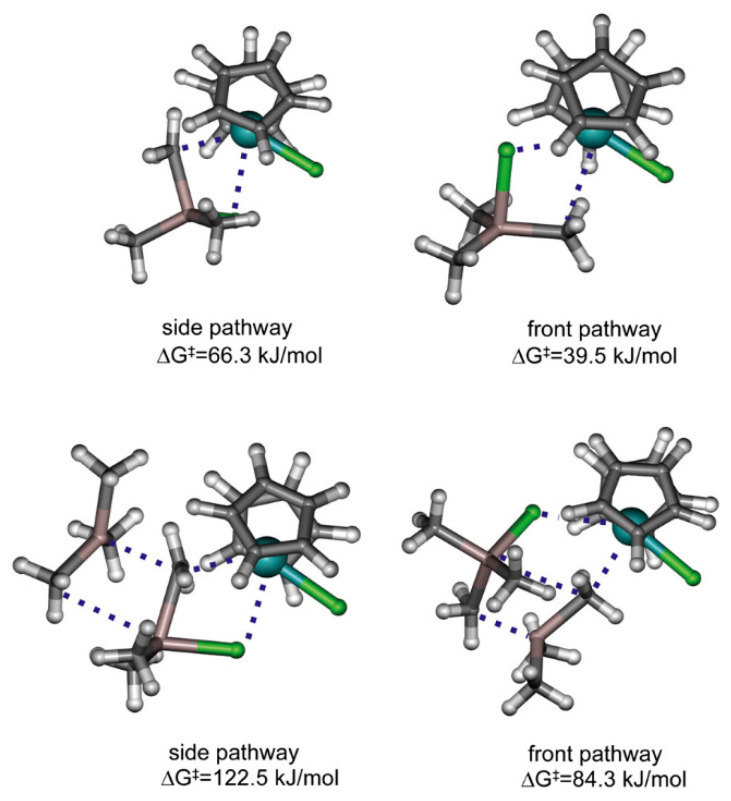
Illustration of transition states for the alkylation of Cp_2_ZrCl_2_ with Me_3_Al (**top**) and Al_2_Me_6_ (**bottom**). Reprinted with permission from [123]. Copyright (2012) Elsevier B.V.

The difference in equilibria constants for this reaction can be explained based on the results of DFT modeling, as shown by Linnolahti et al., who showed that catalyst alkylation step by AlMe_3_ has two viable routes. AlMe_3_ coordinates to the metallocene either from the side or from the front, leading to corresponding alkylation reaction pathways. The kinetically favorable route is dependent on the ligand structure of the catalyst: the front pathway is preferred for Cp_2_ZrCl_2_ (Figure 11) but is sterically hindered for Me_2_C(Cp)(Flu)Zrl_2_, thereby preferring the side pathway [123]. Similar results have also been obtained in a more recent study by Kumawat and Gupta [124]. **Zr01** was also detected by IR spectroscopy during studies of the activation of Cp_2_ZrCl_2_ by MAO [125].

Dimethyl derivatives of zirconocenes are formed during the second stage of the process. The intermediate complex of the formula Cp_2_ZrMeCl⋯AlMe_3_ (**Zr01**) was detected by ^1^H NMR at −85 °C; however, at −60 °C, the characteristic signal of Zr–Me group had already disappeared [126].

**Figure 12:**
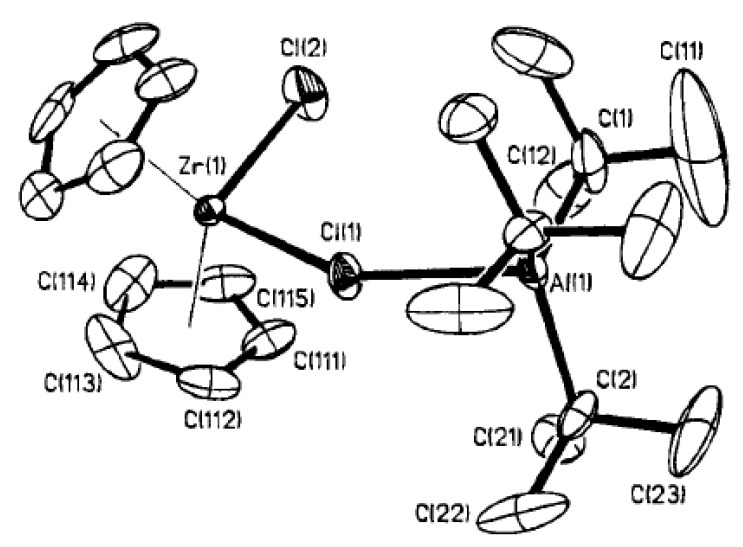
Molecular structure of one of the crystallographic independent molecules of Cp_2_ZrMeCl⋯AlMe_3_ (**Zr01′**). Reprinted with permission from [127]. Copyright (1995) American Chemical Society.

The presence of direct Zr–(μ-Cl)–Al bonding in Cp_2_ZrMeCl complexes with R_3_Al has clearly been proven by Barron et al. [127]. They showed that even Me_2_Al can coordinate with Cl (d(Al–Cl) = 2.46 Å, d(Zr–Cl) = 2.52 Å, Figure 12) with the formation of a relatively stable complex Cp_2_ZrMeCl⋯AlMe_3_ (**Zr01′**).

**Scheme 14:**
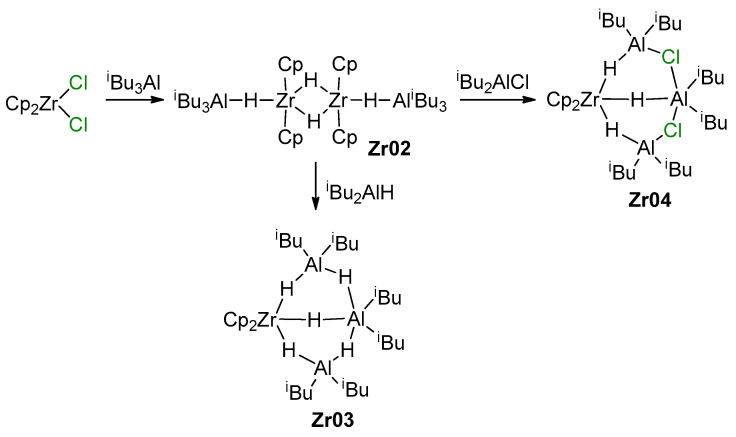
Reaction of Cp_2_ZrCl_2_ with ^i^Bu_3_Al or ^i^Bu_2_AlH [128,129].

It is obvious that the reactions of L_2_ZrCl_2_ with ^i^Bu_2_AlH or ^i^Bu_3_Al may result in the formation of Zr–Al hydrides, which is accompanied by isobutylene elimination in the latter case [128]. The rate and equilibria of the reaction with ^i^Bu_3_Al also strongly depend on the nature of the η^5^-ligand fragment L_2_; for instance, Ph_2_C(Cp)(Flu) forms a mono-alkyl complex Ph_2_C(Cp)(Flu)Zr(^i^Bu)Cl even with a 50-fold excess of ^i^Bu_3_Al, whereas Cp_2_ZrCl_2_ reacts with an excess of ^i^Bu_3_Al with the formation of [Cp_2_ZrH_2_·Al^i^Bu_3_]_2_ (**Zr02**) [128], and reaction with ^i^Bu_2_AlH results in **Zr03** [129] (Scheme 14). In the presence of ^i^Bu_2_AlCl, **Zr02** forms the Zr–Al_3_ complex **Zr04**; ring-substituted bis(cyclopentadienyl) L_2_ZrCl_2_ complexes demonstrate similar behavior [129]. For Zr *ansa*-complexes, the formation of L_2_ZrCl(μ-H)_2_Al^i^Bu_2_ species was detected.

**Scheme 15:**
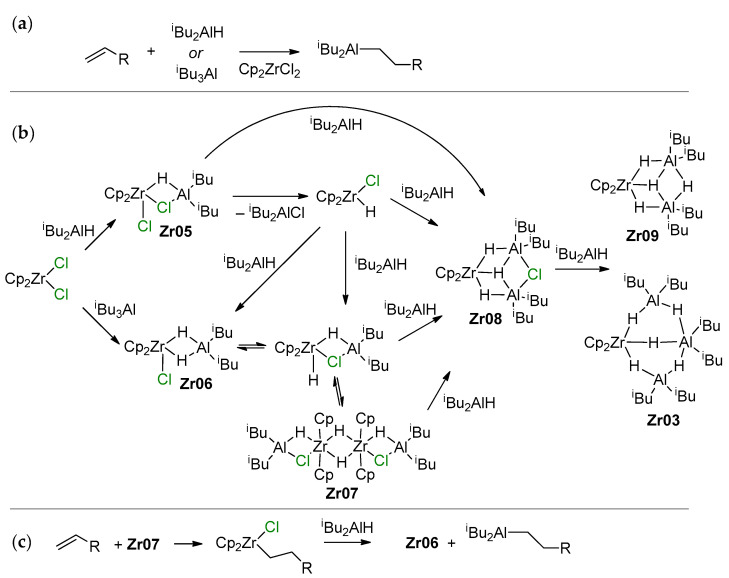
(**a**) Hydroalumination of α-olefins; (**b**) Zr–Al species formed during the reaction of Cp_2_ZrCl_2_ with ^i^Bu_3_Al or ^i^Bu_2_AlH; (**c**) the stages of Cp_2_ZrCl_2_-catalyzed hydroalumination [18,131,132,133].

Efficient Cp_2_ZrCl_2_-catalyzed hydroalumination uses ^i^Bu_2_AlH or its equivalent ^i^Bu_3_Al as Al sources due to the ease of isobutylene elimination with the formation of Al–H bonds (Scheme 15a). A greatly simplified hypothetical mechanism of this reaction, proposed by Negishi [130], was substantially developed and supplemented by new experimental and theoretical results by Dzhemilev and Parfenova group. In [131], they proposed a hydroalumination mechanism involving the intermediate formation of Cp_2_Zr(μ-Cl)(μ-H)Al (**Zr05**) and Cp_2_Zr(μ-H)_2_Al (**Zr06**) species that are able to perform π-coordination and insertion of the α-olefin molecule. In the same study, a dimer of the Zr–Al complex (**Zr07**) was detected, and excess ^i^Bu_3_Al or ^i^Bu_2_AlH resulted in the formation of Zr–Al_2_ and Zr–Al_3_ complex hydrides (**Zr03**, **Zr08**, **Zr09**; the latter may contain μ-Cl fragments) (Scheme 15b). It was later discovered that in comparison with other Cp_2_Zr-based complexes (including the Schwartz’s reagent Cp_2_ZrHCl), **Zr03** demonstrated the highest catalytic activity in hydroalumination (Scheme 15c) [132,133]. Probably, Cl-containing organoaluminum fragments in Zr–Al hydride complexes accelerate the intramolecular exchange between bridging and terminal H atoms and shift the equilibrium between the dimeric and active monomeric forms.

**Figure 13:**
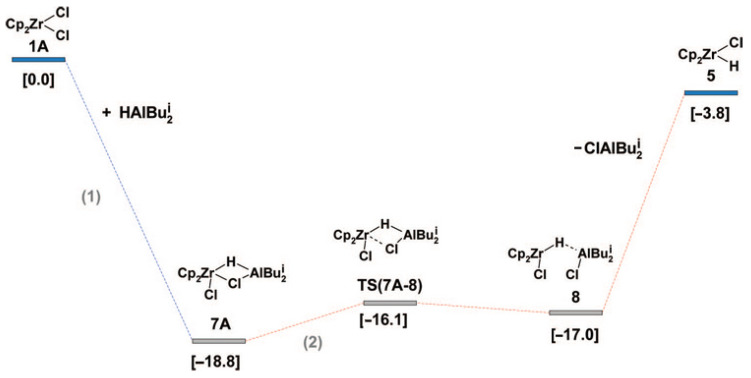
Energy profile of Cp_2_ZrHCl formation (reaction enthalpies in kcal·mol^−1^ are shown in square brackets). Reprinted with permission from [135]. Copyright (2009) American Chemical Society.

The validity of Scheme 15 was confirmed in follow-up studies of the reaction of different L_2_ZrCl_2_ complexes with ^i^Bu_2_AlH [134]; **Zr08**-type complexes were found to be active species of the hydroalumination process. **Zr08** species are formed via Zr–(μ-Cl)–Al intermediate **Zr06′**; therefore, the presence of Cl in the catalytic system is important for the hydroalumination process. These experimental results were in line with the results of a number of theoretical studies on the mechanism of L_2_ZrCl_2_-catalyzed hydroalumination performed by Parfenova et al. In [135], the formation of Cp_2_ZrCl_2_-based catalytic species was analyzed in detail. The main results of the modeling matched Scheme 15; in particular, by the optimization of the molecular structure of **Zr06** that was found to be relatively stable (Figure 13). In accordance with Scheme 15, the kinetic model for the reaction steps was developed [136,137].

In [138], α-olefin interactions with catalytically active centers were studied by DFT and ab initio calculations. It was shown that Cp_2_ZrHCl, **Zr06**, **Zr08**, and **Zr09** are able to perform coordination and insertion of the α-olefin molecule; the activity decreases in the order Cp_2_ZrHCl > **Zr06** > **Zr08** > **Zr09**. The results of the modeling correlate with experimental data on Cp_2_ZrCl_2_-catalyzed hydroalumination, considering the equilibria between **Zr07** (isolated active catalyst) and **Zr06** (highly reactive intermediate) and low solubility of Cp_2_ZrHCl that slows down the reaction. DFT modeling of the final stage of hydroalumination (Zr → Al alkyl transfer) was conducted for Cp_2_ZrCl(*^n^*Pr)–^i^Bu_2_AlX system (X = H, Cl, ^i^Bu) [139]. It was shown that the reaction of ^i^Bu_2_AlH needed no activation energy and proceeded through the coordination of ^i^Bu_2_AlH both outside and inside of the C–Zr–Cl angle, and led to the Cp_2_ZrHCl⋯Al ^i^Bu_2_Pr association, which further dissociated into the Al ^i^Bu_2_Pr and Al^i^Bu_2_*^n^*Pr. Transmetallation in Cp_2_ZrCl(*^n^*Pr)⋯ClAl ^i^Bu_2_ occurs via the coordination of ^i^Bu_2_AlCl to the inside of the C–Zr–Cl angle with the formation of a bridging complex Cp_2_ZrCl(μ-Cl)(μ-*^n^*Pr)Al ^i^Bu_2_, followed by intramolecular ligand exchange that results in Cp2ZrCl_2_⋯Al^i^Bu_2_*^n^*Pr association which further decomposes into Al^i^Bu_2_*^n^*Pr and Cp_2_ZrCl_2_. It was also shown that the transmetallation of the propyl group in Cp_2_ZrCl(*^n^*Pr)⋯Al^i^Bu_3_ is less probable due to the high activation barrier (ΔG^≠^ = 31.9 kcal·mol^−1^). In this way, the transmetallation rates of Cp_2_ZrCl(*^n^*Pr) under the action of ^i^Bu_2_AlX decrease in the order ^i^Bu_2_AlCl > ^i^Bu_2_AlH > ^i^Bu_3_Al [139].

**Scheme 16:**
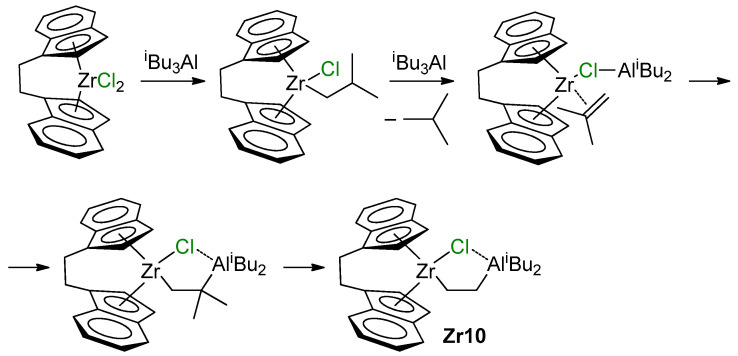
The formation of **Zr10 [140]**.

Current views on the mechanism of the reaction of L_2_ZrCl_2_ with ^i^Bu_3_Al were substantially expanded in the recent study by Conley et al. [140]. When studying reactions of [CH_2_CH_2_(η^5^-C_9_H_6_)_2_]ZrCl_2_ with 12 eq. of organoaluminum compound, they detected the formation of Zr–(μ-Cl)–Al complex **Zr10**, presumably through β-Me elimination/carboalumination stages (Scheme 16). Notably, the π-complex in this Scheme represents the Zr(II) complex.

**Scheme 17:**
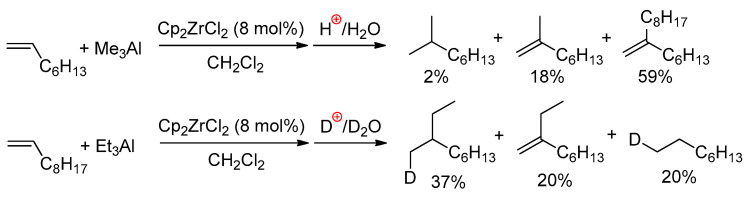
Cp_2_ZrCl_2_-catalyzed carboalumination [143,144].

Cp_2_ZrCl_2_-catalyzed carboalumination was discovered by Van Horn and Negishi [141]. This reaction was also considered in the review by Parfenova [18] and in the more recent review of Negishi [142]. In most studies on catalytic carboalumination, Me_3_Al and (*n*-Alkyl)_3_Al have been used to avoid β-hydride elimination. Cp_2_ZrCl_2_ is not an efficient carboalumination catalyst (Scheme 17) [143,144]. As shown by Parfenova et al., the efficiency of carboalumination can be increased by the rational design of η^5^-ligands in the L_2_ZrCl_2_ pre-catalyst [145].

**Scheme 18:**
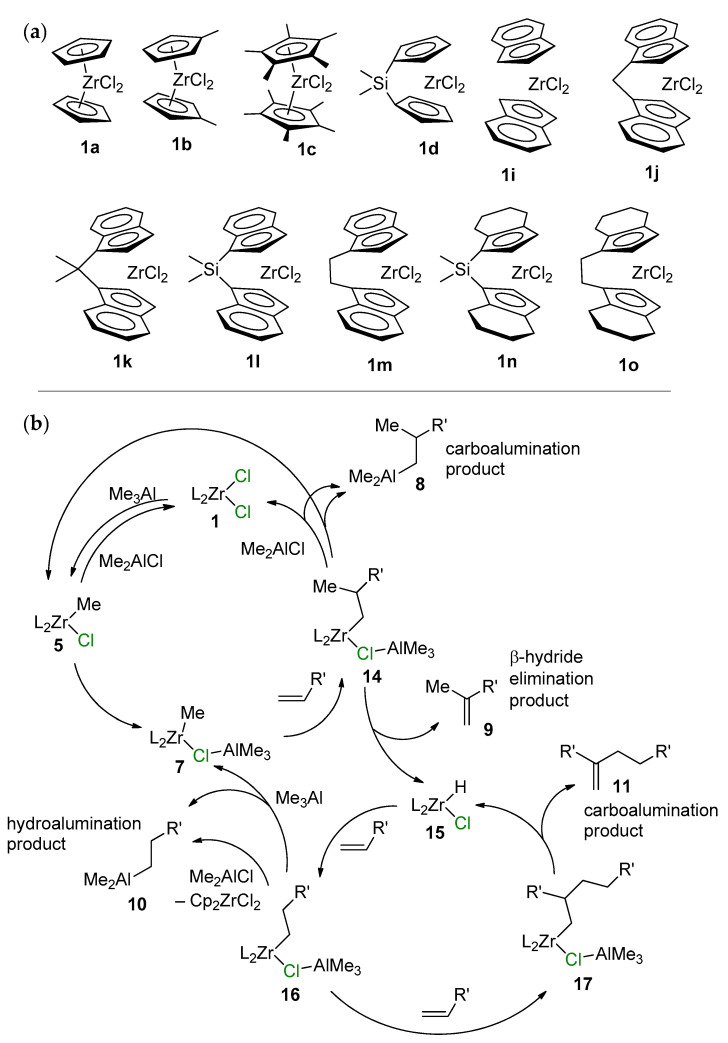
(**a**) Structural formula of the L_2_ZrCl_2_ complexes studied in the catalytic reaction of oct-1-ene with Me_3_Al; (**b**) probable mechanism of the L_2_ZrCl_2_-catalyzed reaction of alkenes with Me_3_Al [126].

When using Me_3_Al as an activator, both carboalumination and hydroalumination processes can take place in the presence of other L_2_ZrCl_2_ complexes. In 2018, Parfenova et al. [126] studied the ligand exchange in 15 different zirconocenes, and the oct-1-ene reactivity of 11 of them (Scheme 18a) for L_2_ZrCl_2_–Me_3_Al systems. Based on the results of NMR spectral studies and catalytic experiments, a mechanism of the L_2_ZrCl_2_-catalyzed reaction of alkenes with Me_3_Al was proposed (Scheme 18b). In the first step, complex 1 reacted with Me_3_Al to yield L_2_ZrClMe (5). The subsequent reaction of complex 5 with Me_3_Al yielded intermediate 7 (**Zr01**, see above), in which the Zr–Me bond is more polarized due to associations with electron-deficient Me_3_Al. As shown above, complex **Zr01** exists in the associated state at temperatures below 230 K. At room temperature, it dissociates to Cp_2_ZrMeCl and Me_3_Al and its concentration becomes negligibly low; thus, the alkene does not react. Switching to the catalytic reaction shifts the equilibrium towards **Zr01**, and products 8–11 are formed in the system.

Notably, Cp_2_ZrMe_2_ demonstrated low catalytic activity in the reaction with oct-1-ene and Me_3_Al. Complex 6 is not formed in the reaction of Cp_2_ZrCl_2_ with Me_3_Al; thus, a key role is played by **Zr01**-type intermediates. The degree of association of L_2_ZrMeCl with Me_3_Al also depends on the π-ligand environment of Zr and the nature of the solvent. In this way, realization of the process presented in Scheme 18b requires the presence of Cl in the reaction mixture. In other words, the presence of the Zr–(μ-Cl)–Al structural fragment in key catalytic species and the alkene insertion would, most likely, be accelerated in the L_2_ZrMe(μ-Cl)AlMe_3_ active complex. 

Catalytic reactions were conducted in two different solvents (CH_2_Cl_2_ and toluene) using a L_2_ZrCl_2_/alkene/AlMe_3_ ratio of 1:50:60. The results of the experiments are presented in Table 1.

**Scheme 19:**
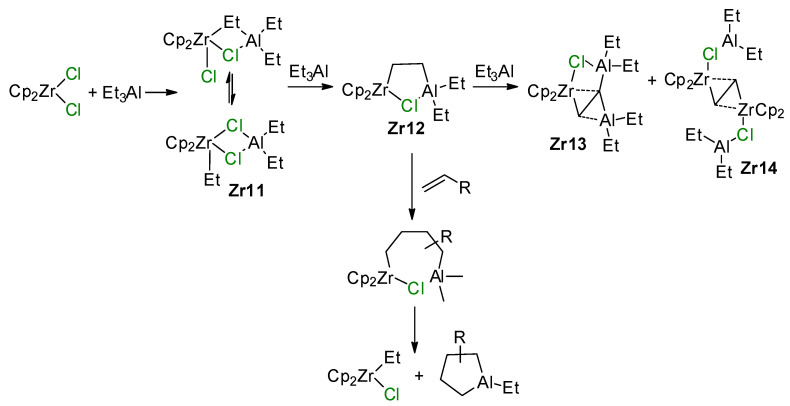
Reaction of Cp_2_ZrCl_2_ with Et_3_Al [146] and the cycloalumination process [148].

**Figure 14:**
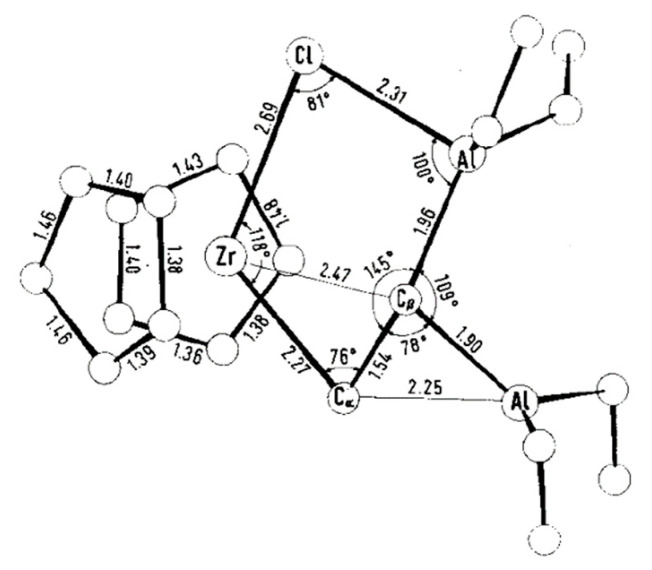
Molecular structure of **Zr13**. Reprinted with permission from [147]. Copyright (1974) Wiley-VCH Verlag GmbH & Co.

The reaction between Et_3_Al and Cp_2_ZrCl_2_ has a complex mechanism, which occurs in several elementary steps [146]. When using 1 eq. of Et_3_Al, the Zr–(μ-Cl)–Al complex **Zr11** is formed. With an excess of Et_3_Al, C–H activation takes place to yield a relatively unstable species, **Zr12**, which is subsequently converted to a more stable **Zr13** via the secondary C–H activation along with a smaller amount of **Zr14** (Scheme 19). The molecular structure of **Zr13** was determined by XRD [147] (Figure 14). In the presence of α-olefin, the highly reactive species **Zr12** is further transformed into a cycloalumination product [148]. The mechanism of this reaction was studied by DFT modeling, which confirmed the importance of Zr–(μ-Cl)–Al bonding for all key reaction stages [149,150,151,152].

**Scheme 20:**
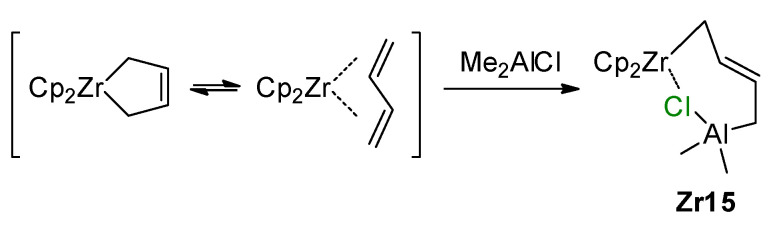
Reaction of the Zr(IV) buta-1,3-diene complex with Me_2_AlCl [153].

An interesting complex, **Zr15** with a Zr–(μ-Cl)–Al fragment, was formed as a result of the reaction of the Cp_2_Zr buta-1,3-diene complex with Me_2_AlCl (Scheme 20) [153].

**Figure 15:**
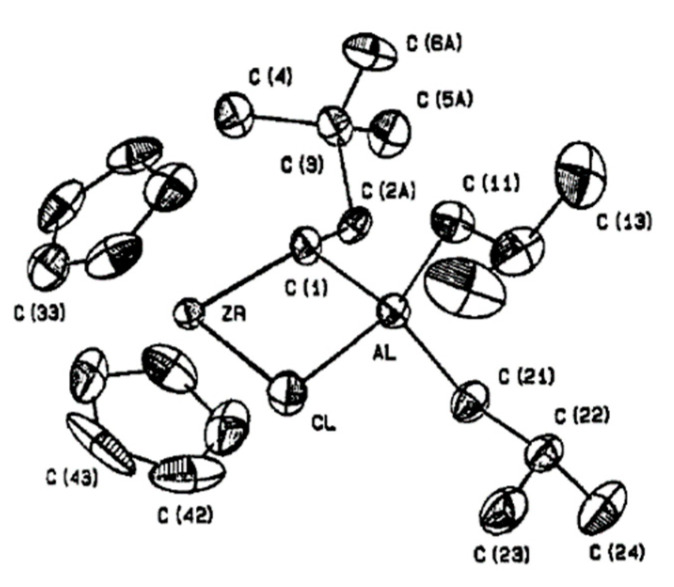
Molecular structure of complex **Zr16**. Reprinted with permission from [155]. Copyright (1987) American Chemical Society.

Zr analogs of the Tebbe reagent Cp_2_Zr(μ-CHR)(μ-Cl)AlR′_2_ were obtained (a) by the reaction of Cp_2_Zr(Cl)CH=CHR with R′_2_AlH or (b) by the reaction of Cp_2_ZrHCl with R′_2_AlCH=CHR [154,155]. The molecular structure of one of these complexes with R = CH_2_*^t^*Bu and R′ = ^i^Bu (**Zr16**) was proven by XRD (Figure 15). Similar complexes have not demonstrated any synthetic or catalytic prospects.

### 3.2. Zirconocene Cationic Complexes in Oligomerization and Polymerization of α-Olefins

**Scheme 21:**
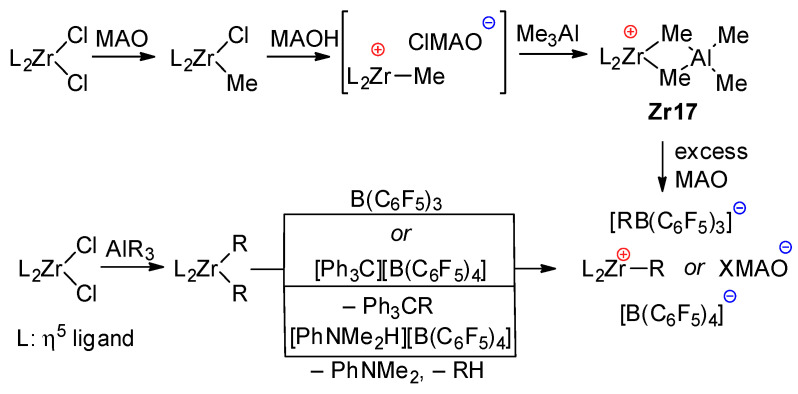
Conventional mechanism of L_2_ZrCl_2_ activation by MAO and perfluoroaryl borates.

Generally accepted mechanisms of the activation of zirconocene pre-catalysts L_2_ZrCl_2_ depend on the type of activator. When using MAO, methylation results in a L_2_ZrMeCl complex, which eliminates Cl^−^ under the action of MAO. However, when using a slight excess of MAO, Me_3_Al (inevitably present in MAO due to its dynamic nature), the intermediate active cation forms the inactive cationic complex **Zr17**, which can be reactivated when using a large excess of MAO. Notably, MAO has limited ability to activate L_2_ZrCl_2_; the nature of the primary η^5^-ligand environment (L_2_Zr fragment) affects the rate and equilibria of the formation of the catalytic species [122,156]. In the presence of trialkylaluminum and perfluoroaryl borates, an alkylzirconocene cation is formed through the alkylation of L_2_ZrCl_2_ with AlR_3_, followed by the elimination of one Zr–alkyl under the action of B(C_6_F_5_)_3_ or by the alkyl abstraction or protonation of Zr–alkyl by [Ph_3_C]^+^ or [PhNMe_2_H]^+^ counterions of [B(C_6_F_5_)_4_]^−^, respectively (Scheme 21).

**Figure 16:**
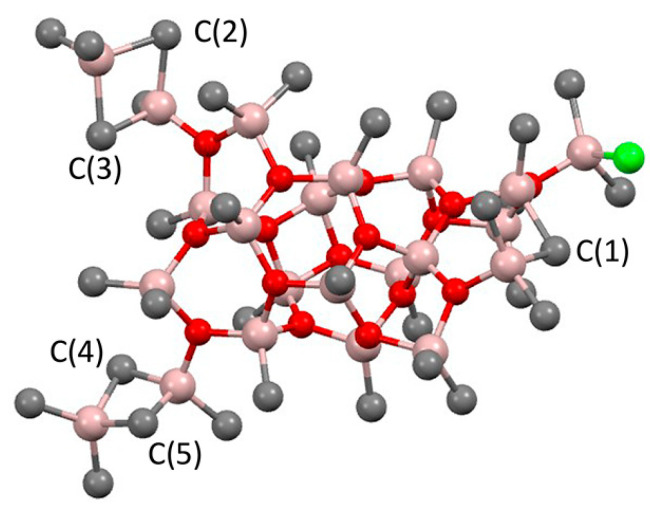
Model for anion with *m*/*z* 1395 featuring a terminal Me_2_AlCl. Reprinted with permission from [28]. Copyright (2017) American Chemical Society.

The negative role of Cl^−^ in the zirconocene-catalyzed polymerization of α-olefins when using MAO as an activator was demonstrated by Cramail et al. in the framework of the simplified MAO model [157]; however, further studies have shown that not everything is so unambiguous, primarily because of the incompleteness of the early conceptions of the MAO structure. In recent years, this issue was clarified; essentially, the presence of Cl-containing MAO species as counter-ions was confirmed experimentally [28,125,158,159], In particular, the molecular ion with an *m*/*z* ratio of 1395 (Figure 16) was detected when analyzing the activation of Cp_2_ZrCl_2_ by MAO. Evidently, it is the Al–Cl fragment in this molecule that can coordinate at the Zr catalytic center. This model of ‘chlorinated’ MAO was developed on the basis of previous mass spectrometry studies [26].

In [23], Linnolahti et al. proposed a simple and visual model of the structure of an MAO cluster, suitable for DFT modeling. They demonstrated that the chlorination of MAO leads to the overall facilitation of catalyst activation processes.

**Figure 17:**
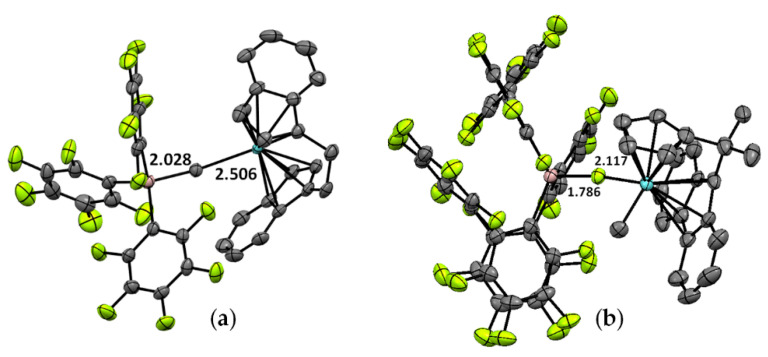
Molecular structures of the complexes (**a**) {[(CH_2_CH_2_)Ind_2_]ZrMe}^+^[MeAl(C_6_F_5_)_3_]^−^ (**Zr18**) [160] and (**b**) [Me_2_C(Cp)(Flu)]ZrMe]^+^[FAl(2-C_6_F_5_C_6_F_4_)_3_]^−^ (**Zr19**) [22].

There are no examples of cationic *catalytically active* zirconocene species with Zr–(μ-Cl)–Al fragments whose molecular structures have been proven by XRD. It is worth pointing out here that cationic Zr complexes with [MeAl(2-C_6_F_5_C_6_F_4_)_3_]^−^ (**Zr18**) [160] and [FAl(2-C_6_F_5_C_6_F_4_)_3_]^−^ ions (**Zr19**) [22] have been separated and analyzed by XRD. As can be seen in Figure 17, the differences between Zr–(μ-X) and Al –(μ-X) are equal to 0.48 and 0.33 Å for X = C and X = F, respectively. Whether Al(C_6_F_5_)_3_ is capable of Cl abstraction from L_2_CpCl(Alkyl) complexes is now an open question.

In recent publications [161,162], the mechanism of the activation of L_2_ZrCl_2_ pre-catalysts by MAO was significantly revised with new experimental data that point to the intermediate formation of [L_2_Zr(μ-Cl)_2_AlR_2_]^+^ cationic species via the generation of R_2_Al^+^, followed by their reaction with L_2_ZrCl_2_. At the same time, an alternative activation pathway was proposed for the reaction of L_2_ZrCl_2_ with [^i^Bu_2_Al][B(C_6_F_5_)_4_], namely, Cl^−^ abstraction with a formation of L_2_ZrCl^+^ species [33].

**Figure 18:**
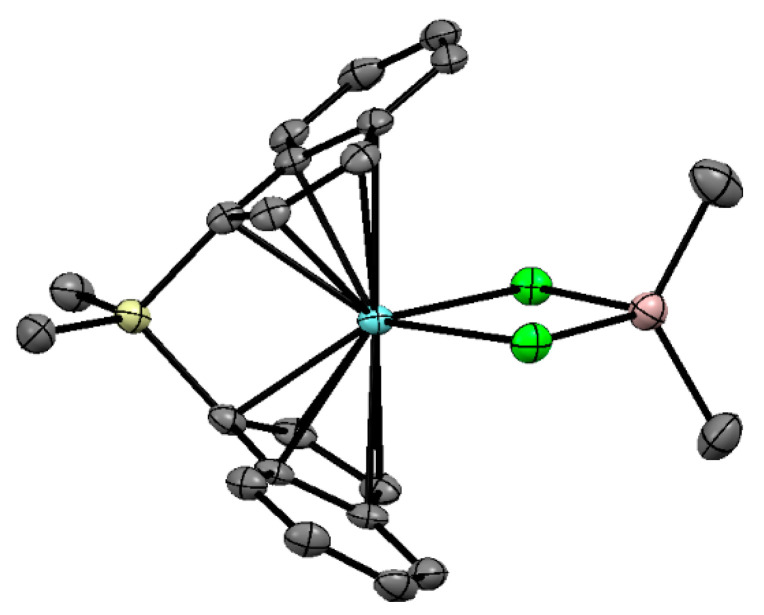
Molecular structure of *rac*-[Me_2_Si(η^5^-C_9_H_6_)_2_]Zr(μ-Cl)_2_AlMe_2_ (**Zr22**). Reprinted with permission from [164]. Copyright (2013) American Chemical Society.

Notably, the cationic complex {*rac*-[Me_2_Si(η^5^-C_9_H_6_)_2_]Zr(μ-Cl)_2_Al^i^Bu_2_}^+^ (**Zr20**) was formed by the reaction of {*rac*-[Me_2_Si(η^5^-C_9_H_6_)_2_]Zr(μ-H)_3_(Al^i^Bu)_2_}^+^ and characterized by NMR spectroscopy [163]. The complex {*rac*-[Me_2_Si(η^5^-C_9_H_6_)_2_]Zr(μ-Cl)_2_AlMe_2_}^+^ (**Zr21**) was similarly obtained by the reaction of {*rac*-[Me_2_Si(η^5^-C_9_H_6_)_2_]Zr(μ-Cl)_2_AlMe_2_}^+^ with Me_3_AlCl [164]. XRD studies of type [L_2_Zr(μ-Cl)_2_AlR_2_]^+^ complexes are mentioned in [33], referring to conference papers [165,166]; however, the Cambridge Crystallographic Data Centre (CCDC) has no information about these compounds. The structure of {*rac*-[Me_2_Si(η^5^-C_9_H_6_)_2_]Zr(μ-Cl)_2_AlMe_2_}^+^ was proven by a combination of chemical modification and XRD analysis of the Zr(III) neutral complex *rac*-[Me_2_Si(η^5^-C_9_H_6_)_2_]Zr(μ-Cl)_2_AlMe_2_ (**Zr22**) obtained by the reduction of **Zr21** (Figure 18). The complexes **Zr20**–**Zr22** were inactive in α-olefin polymerization.

**Scheme 22:**
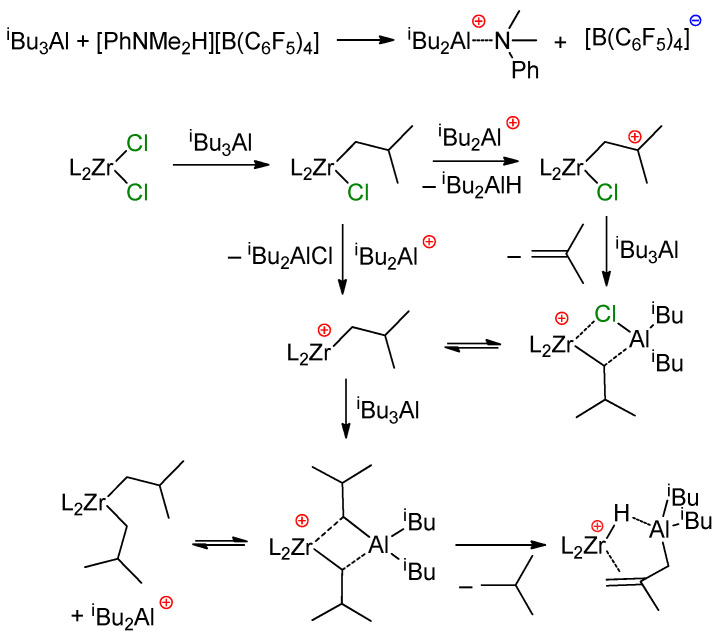
Proposed formation of [Ph_2_C(Cp)(Flu)Zr(μ-H)(μ-C_4_H_7_)Al^i^Bu_2_]^+^ [128].

In the presence of [Ph_3_C][B(C_6_F_5_)_4_], the activation of L_2_ZrCl_2_ by ^i^Bu_2_AlH results in the formation of complex cationic hydrides L_2_Zr(μ-H)_3_(Al^i^Bu_2_)_2_^+^ via a L_2_ZrCl(μ-H)_2_Al^i^Bu_2_ intermediate [163]. However, the reaction of L_2_ZrCl_2_ (L_2_ = Ph_2_C(Cp)(Flu)) with TIBA and a [PhNMe_2_H][B(C_6_F_5_)_4_] activator proceeds in a complex pathway, allegedly involving Zr–(μ-Cl)–Al species (Scheme 22) [128].

**Scheme 23:**
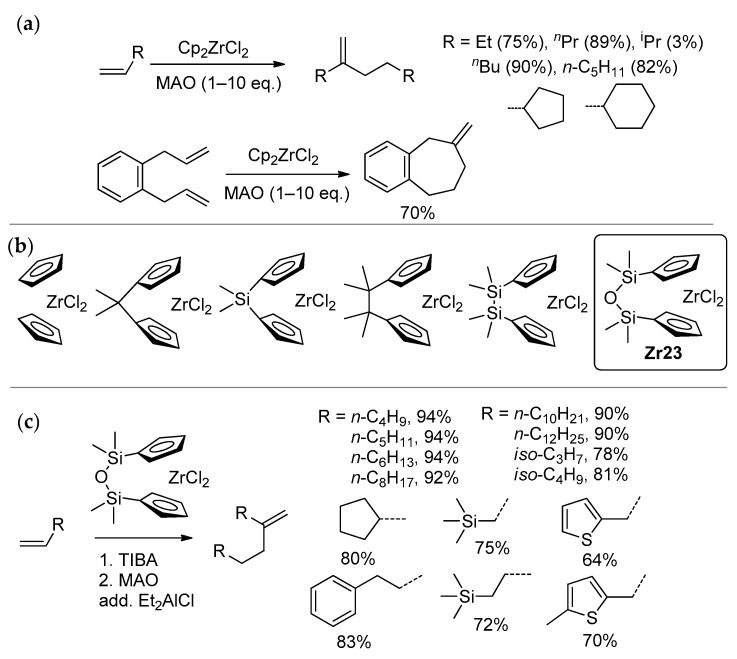
(**a**) Zirconocene-catalyzed selective dimerization of α-olefins [38]; (**b**) L_2_ZrCl_2_ catalysts studied in the dimerization of hex-1-ene [39,181]; (**c**) high efficiency of complex **Zr23** in dimerization [40].

Zirconocene-catalyzed selective dimerization of α-olefins (Scheme 23) represents an interesting and practically important catalytic process, for which the participation of Zr–(μ-Cl)–Al is quite possible. Discovered in 1987 by Slaugh and Schoenthal [167], and first published in scientific periodicals by Christoffers and Bergman [37,38], this reaction was further studied by Erker [168], Janiak [169,170], Kissin [171,172], and Longo [173]. The main dimer of undec-1-ene was obtained by Samela et al. using MAO-activated Cp_2_ZrCl_2_ [174], but the reaction products were mistakenly recognized as undec-1-ene trimers. Methylenealkanes can be used in the synthesis of single-component poly-α-olefin oil basestocks [171,175,176] and other value-added chemicals [169,171,172,177,178,179,180].

By the mid-2010s, only Cp_2_ZrCl_2_ [37,38] and [Me_2_Si(η^5^-C_5_H_4_)_2_]ZrCl_2_ [173] were efficiently used as pre-catalysts in selective α-olefin dimerization. In comparative studies of a series of Zr complexes, activated sequentially by ^i^Bu_3_Al and MAO, in the dimerization of hex-1-ene (Scheme 23b) Nifant’ev et al. established the formula of complex **Zr23** [39,181] that demonstrated high selectivity and activity in the dimerization of different α-olefins, including sterically hindered substrates [40]. Notably, the selectivity of dimerization increased with a decrease in the dihedral angle between cyclopentadienyl rings. During these experimental studies, increases in the selectivity of dimerization when adding R_2_AlCl were detected. A similar ‘chlorine effect’ was previously demonstrated by the chemists Idemitsu Kosan [182,183], and later by Parfenova et al. [184]. As was shown by Parfenova’s group, Zr_2_ bimetallic species are also active in selective dimerization [185]; however, the use of these in situ forming species is not of interest in practice.

**Scheme 24:**
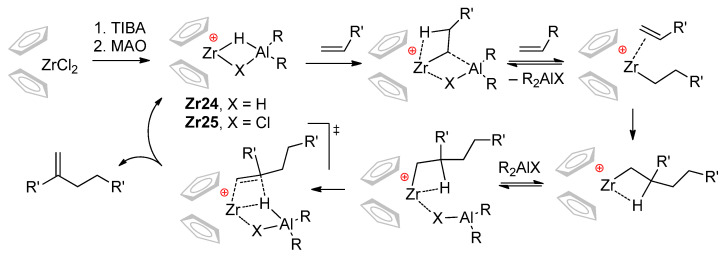
Mechanistic concept explaining the selective formation of α-olefin dimers by Zr–(μ-Cl)–Al bonding (X = H, Cl) [39,40,41,42].

The ‘chlorine effect’ was also noted by Christoffers and Bergman [38]; they suggested the retention of Cl at the Zr catalytic center (Cp_2_ZrR^+^⋯ClMAO^−^ species) on the one hand, but on the other, proposed Cp_2_ZrH^+^ as the actual catalyst. Based on experimental results, Nifant’ev and Ivchenko proposed a new mechanistic hypothesis, expanding the scope of the conventional cationic mechanism of the zirconocene-catalyzed oligomerization of α-olefins [39,40]. This hypothesis implies the participation of Zr–Al catalytic species **Zr24** and **Zr25** capable of reversible insertion of the α-olefin molecule. After the second α-olefin insertion, the reversible coordination of R_2_AlX fragment can facilitate irreversible β-hydride elimination with the formation of methylenealkanes that are inert towards **Zr24** and **Zr25** (Scheme 24). It was also supposed that R_2_AlCl should demonstrate the best efficiency as a selective ‘limiter’ of the degree of polymerization (*DP*_*n*_) to 2.

**Figure 19:**
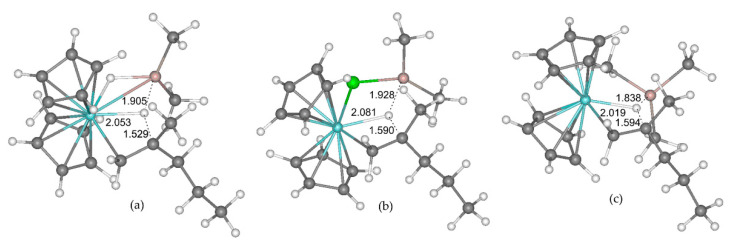
Calculated geometries of the transition states of β-hydride elimination in Cp_2_Zr(µ-X)(CH_2_CHMePr)AlMe_2_ model complexes. (**a**) X = H; (**b**) X = Cl; (**c**) X = Me. The distances Zr–H, Al–H and βC–H are specified (Å). Reprinted with permission from [41]. Copyright (2019) MDPI.

These hypotheses were supported by additional experiments and DFT calculations [41,42]. In [41], oligomerization of hex-1-ene with the use of Cp_2_ZrCl_2_ pre-catalyst was studied experimentally, and DFT optimizations for all possible reaction pathways of prop-1-ene oligomerization with and without the involvement of R_2_AlX (R = Me, ^i^Bu; X = H, Cl, Me) were performed. The key stage of the α-olefin formation was chain termination after insertion of the second α-olefin molecule. For the simple cationic model, this process is carried out on the mechanism of β-hydride transfer to monomer, whereas with the assistance of the R_2_AlX β-hydride, elimination occurs. In the latter case, Al atom demonstrates a cooperative effect (Figure 19). For R = Me, the values of the activation barriers ΔG^≠^ of β-hydride elimination stage were 17.7 (H), 13.6 (Cl), and 16.2 (Me) kcal/mol. Thus, β-hydride elimination is the most affected by Me_2_AlCl coordination at the Zr atom.

**Figure 20:**
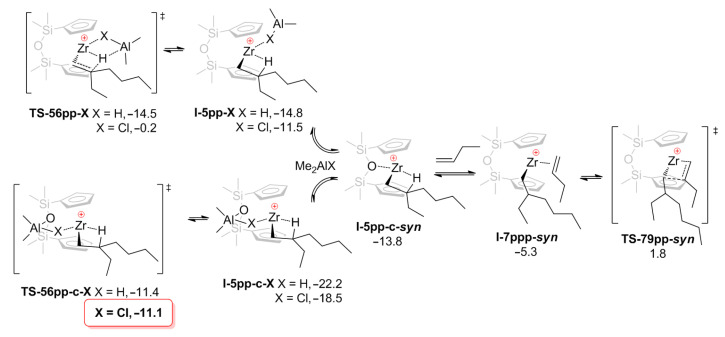
Transformations of O[SiMe_2_(η^5^-C_5_H_4_)]_2_Zr–derived β-agostic complex **I-5pp** with the participation of Me_2_AlX species. The values of calculated free energies (kcal/mol) relative to **I-2pβ-c** are given. Reprinted with permission from [42]. Copyright (2020) MDPI.

**Figure 21:**
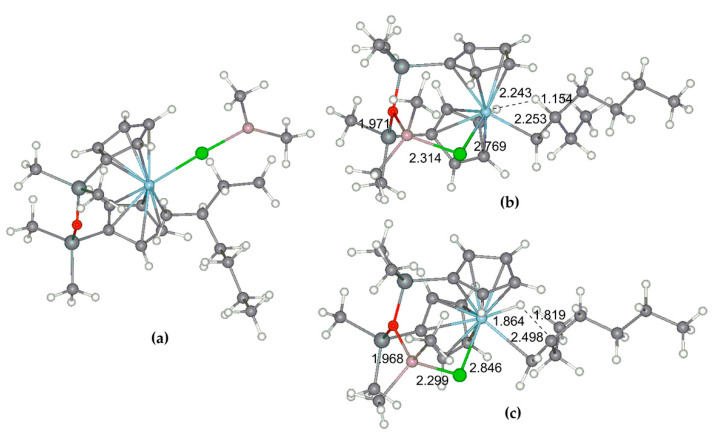
Optimized geometries of O[SiMe_2_(η^5^-C_5_H_4_)]_2_Zr–derived stationary points **I-5pp-Cl** (**a**), **I-5pp-c-Cl** (**b**), and transition state **TS-56pp-c-Cl** (**c**). Reprinted with permission from [42]. Copyright (2020) MDPI.

In the study by Nifant’ev et al. [42], oct-1-ene oligomerization with the use of pre-catalysts Cp_2_ZrCl_2_, Cp_2_ZrMe_2_, O[SiMe_2_(η^5^-C_5_H_4_)]_2_ZrCl_2_ (**Zr23**), and its dimethyl derivative **Zr23′** was studied, DFT modeling was conducted for both zirconocenes using but-1-ene as a model α-olefin. The main theoretical results with regard to Cp_2_Zr-derived species were in line with the results of prior research [41], whereas in the case of **Zr23**-based catalytic species, additional Zr–O and Al–O interactions played a significant role in the catalytic process, stabilizing the reaction intermediates and lowering the activation barriers (Figure 20 and Figure 21).

**Scheme 25:**
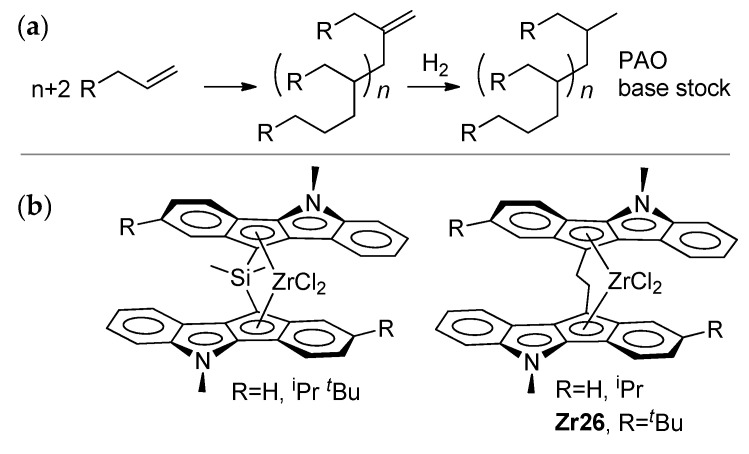
(**a**) Coordination oligomerization of α-olefins; the idealized uniform molecular structure of the oligomers was achieved when using ‘heterocene’ catalysts (**b**) [43].

Thus, if zirconocene-catalyzed dimerization requires the a minimal excess of MAO (less than 10 eq.) and zirconocenes of the cyclopentadienyl type (Cp_2_ZrCl_2_, **Zr23**), the catalytic oligomerization of α-olefins (Scheme 25a) is usually conducted at higher Al_MAO_/Zr ratios. However, these ratios are hardly capable of the complete ‘fixation’ of R_2_AlCl (MAO as a ‘sponge’ for organoaluminum compounds in the reaction mixture). On the other hand, the greater diversity of zirconocenes used in this reaction still attracts researchers’ attention [17,186,187,188,189,190,191,192]. The activation of L_2_ZrCl_2_ by R_3_Al and perfluoroaryl borates was also found to be efficient in α-olefin oligomerization. This type of activation was used in the recent study by Nifant’ev et al., who reported that –CH_2_CH_2_– bridged indeno[1,2-*b*]indole *ansa*-complexes (Scheme 25b) demonstrate high efficiency in the synthesis of lightweight oligomers of dec-1-ene (*DP*_*n*_ = 3–5) with uniquely homogeneous molecular structures [43].

Such catalytic behavior differs from both the activity and selectivity of conventional zirconocenes; therefore, additional research was carried out. Surprisingly, the addition of ^i^Bu_3_Al to the solution of **Zr26** did not result in Cl → ^i^Bu exchange at the Zr atom. One can assume that the dissolution of **Zr26** occurs through the formation of the **Zr26**–^i^Bu_3_Al complex with weak Cl–Al coordination. Notably, a similar complexation of L_2_ZrCl_2_ with metal alkyls was recently discussed by Kumawat and Gupta in their study on the DFT modeling of zirconocene activation and chain transfer [124].

**Figure 22:**
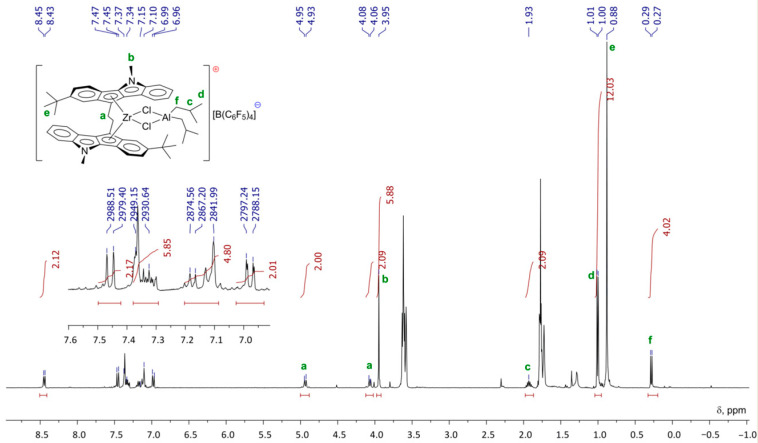
^1^H NMR spectra (400 MHz, THF-d_8_, 20 °C) of **Zr27**. Reprinted with permission from [43]. Copyright (2022) Elsevier B.V.

**Figure 23:**
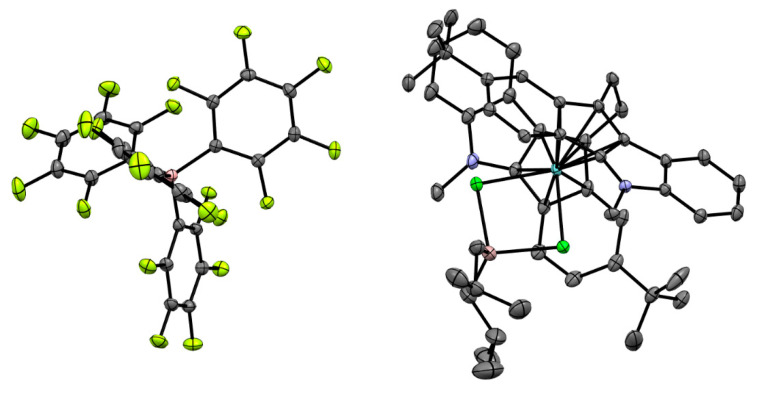
Schemes follow the same formatting. Reprinted with permission from [43]. Copyright (2022) Wiley-VCH Verlag GmbH & Co. Copyright (2016) Royal Society of Chemistry. Copyright (2012) American Chemical Society. Copyright (2015) Springer Nature. Copyright (2019) Elsevier B.V.

At the first stage of the activation, **Zr26** does not exhibit substantial conversion to Zr–Al hydrides under the action of TIBA. However, after the addition of [PhNHMe_2_][B(C_6_F_5_)_4_], a rapid reaction proceeded with the sedimentation of oily low-soluble product. After the addition of dec-1-ene, no oligomerization was observed. Rapid oligomerization was detected when the activation of **Zr26** by TIBA and borate were conducted in the presence of molecular hydrogen, and light-brown crystals of **Zr27** were formed at the end of the reaction. After the addition of dec-1-ene to the reaction mixture, oligomerization started again. It turned out that **Zr27** alone was inactive in dec-1-ene oligomerization in the presence of H_2_; however, when TIBA was added to the **Zr27** suspension in toluene, the rapid oligomerization of dec-1-ene occurred. The final product of the catalyst transformation of **Zr27** was separated and characterized by NMR (Figure 22) and XRD analysis (Figure 23). The spectral view of **Zr27** remained unchanged after 7 days, which indicates high stability of the cationic complex in the solvating solvent (THF).

The experimental fact of the ‘recovery’ of Zr–(μ-Cl)_2_–Al complex **Zr27** clearly indicates that the mechanism of heterocene-catalyzed oligomerization went beyond the conventional cationic mechanism of the zirconocene-catalyzed polymerization of α-olefins. There is a very realistic chance that it is the retention of Zr–Cl–Al coordination that provides structural homogeneity of dec-1-ene oligomers when using heterocene catalysis.

Comparison of the hydrogen response during ethylene polymerization, demonstrated by dichloro- and dimethyl *ansa*–complexes [(η^5^-C_9_Me_6_)SiMe_2_(η^5^-C_5_H_4_)]ZrX_2_ (X = Cl, **Zr28**; X = Me, **Zr29**), immobilized on solid MAO, may also be seen as indirect evidence of Zr–(μ-Cl)–Al bonding in catalytic species: **Zr28**-based catalysts turned out to be inert to molecular hydrogen [193]. The relative stability of Zr–(μ-Cl)–Al bonding in catalyst precursor, L_2_Zr(R)-(μ-Cl)-AlR’_3_ complexes, essentially depends on the electrophilicity of the Al atom. With the introduction of the electron acceptor fragments, e.g., when using ^i^Bu_2_Al(OC_6_F_5_) [194], an ion pair is easily formed, as evidenced by polymerization experiments.

On the basis of the latest investigations [33,43,161,162], it can be concluded that the difference in the mechanisms of the L_2_ZrCl_2_ + AlR_3_ reactions in the absence and presence of MAO (or perfluoroaryl borates) consists of various degrees of ‘chlorination’ for the first-stage products. In the absence of MAO or borate, alkyl transfer from Al to Zr occurs with the formation of LZrR_2_⋯ClAlR_2_ complexes with weak Zr⋯Cl bonds (if R = ^i^Bu, the transformation to Zr–Al hydrides results in heterometallic complexes with stronger Zr–(μ-H)–Al bonds). When using MAO or perfluoroaryl borates, resulting R_2_Al^+^ species rapidly react with LZrCl_2_ to form cationic [LZr(μ-Cl)_2_AlR_2_]^+^, thus creating certain preconditions for the involvement of R_2_AlCl in further reactions with α-olefins.

## 4. Complexes of V

**Scheme 26:**
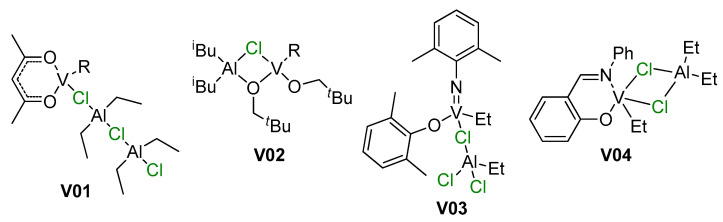
Examples of V–(μ-Cl)–Al active species mentioned in previous reviews [44,195,199].

Vanadium-catalyzed α-olefin polymerization was previously reviewed by van Koten et al. in 2002 [195]; by Gambarotta in 2003 [196]; by Redshaw in 2010 [197]; by Wu and Li in 2011 [198]; by Nomura and Zhang in the same year [199]; and by Phillips et al. in 2020 [44]. In these reviews, the focus was on different types of the coordination compounds of V, pre-catalysts of α-olefin polymerization. The mechanisms of polymerization in these reviews were fragmented; however, in contrast to reviews on group 4 metal Ziegler–Natta and single-site catalysts, the importance of V–(μ-Cl)–Al bonding in pre-catalysts and in catalytic species was not ignored (Scheme 26). Here, in strong contrast to Ti- and Zr-based pre-catalysts, a large number of V-based catalysts are effectively activated by R_2_AlCl of RAlCl_2_, as opposed to ineffective R_3_Al and MAO. In this section, we discuss several examples of V–(μ-Cl)–Al complexes that have been missed in previous reviews, or that had been served poorly, as well as new examples of these complexes.

**Scheme 27:**
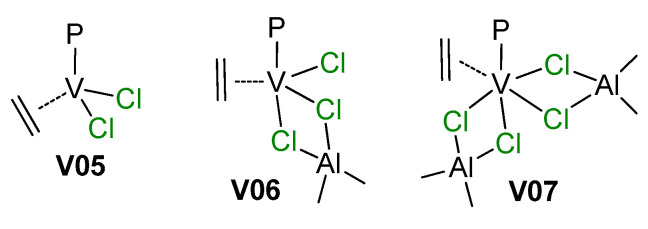
The hypothetical active species for V catalysts (P = polymer chain) [200].

Among older publications on the subject of V-catalyzed polymerizations of α-olefins, the feature article of Zambelli et al. [200] merits special attention. The authors reinvestigated three models of catalytic complexes **V05**–**V07** proposed in the literature for VCl_3_/R_2_AlCl catalyst systems (Scheme 27). DFT modeling of the insertion of ethylene molecule was conducted for P = Me according to the conventional Cossee–Arlman scheme. Thermodynamic data for ethylene insertion were close for all three complexes, but significant differences were observed for values of the activation barrier of insertion that were 16, 16, and only 1.7 kcal·mol^−1^ for **V05**, **V06**, and **V07**, respectively. DFT modeling of prop-1-ene insertion explained the observed syndiotacticity of prop-1-ene polymerization.

**Scheme 28:**
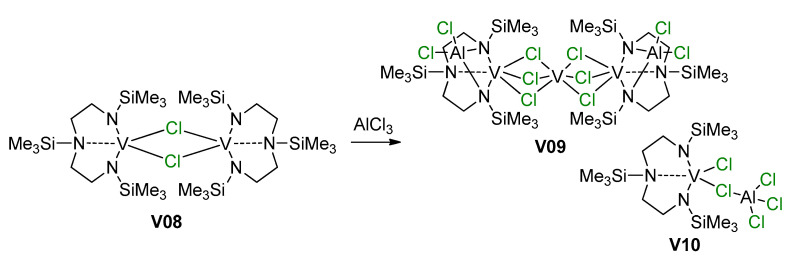
Disproportionation of V(III) complex **V08** [201].

**Figure 24:**
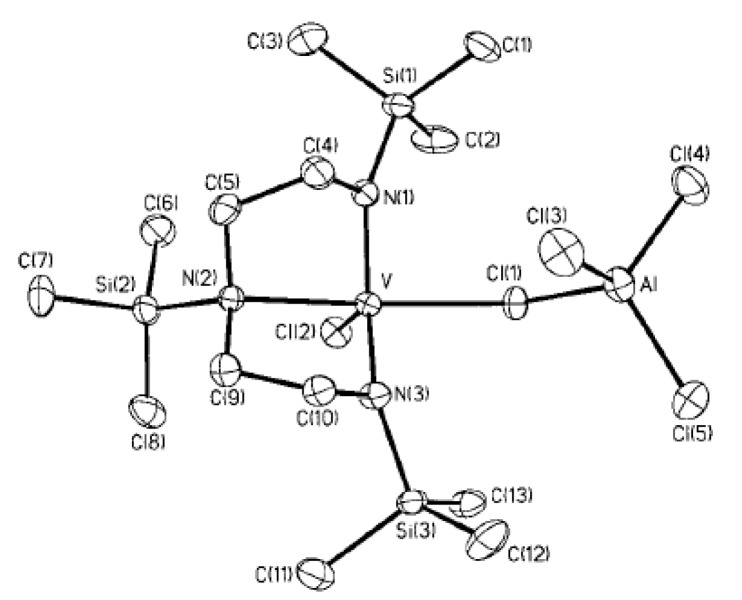
ORTEP plot of **V10**. Thermal ellipsoids are drawn at the 30% probability level. Reprinted with permission from [201]. Copyright (2002) American Chemical Society.

As can be seen in Scheme 26 and Scheme 27, V(III) species are active in polymerization. Obviously, the mechanisms of the deactivation of V centers are important for the development of efficient catalysts. It is thought that the most likely route for deactivation would be reactions with the organoaluminum compound. However, in 2002, Gambarotta et al. demonstrated an alternative pathway of such deactivation via disproportionation of the V(III) complex **V08** with the formation of V(II)/V(III) (**V09**) and V(IV) (**V10**) species [201] (Scheme 28, Figure 24); AlCl_3_ in this process acts as Lewis acid and it cannot be ruled out that organoaluminum co-catalysts can promote catalyst deactivation in a similar way.

**Scheme 29:**
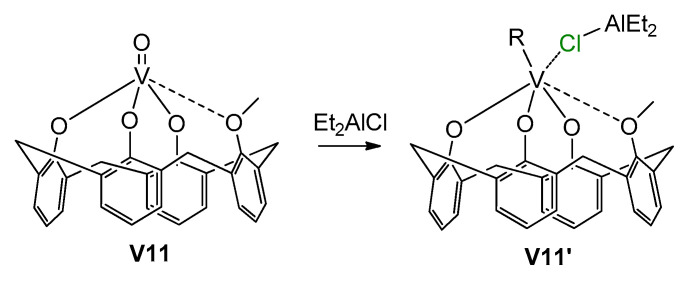
Formation of active V(IV) phenolate complex **V11′** [204].

Another important aspect of V catalysis in α-olefin and diene polymerization was demonstrated by Centore et al. [202], who studied the catalytic activity of the pre-catalyst [(κ^1^-^i^PrN=C(Ph)N^i^Pr)V(O)Cl(μ-Cl)]_2_ using different organoaluminum activators. They showed that the amidinate ligand can easily be removed from the vanadium in the reaction with organometallic cocatalysts, and the active species are closely related to those obtained from other vanadium precatalysts such as VCl_4_ (for example, **V07**). This result imposes certain restrictions on the ligand-oriented design of V-based polymerization catalysts. Stronger V–ligand bonding, such as in **V03** [203], provides a retention of the base ligand environment. Polyphenolate ligands can also provide stability of the catalytic species [204]; in particular, activation of the pre-catalyst **V11** (Scheme 29) resulted in the formation of V(IV) complex **V11′,** which is active in ethylene polymerization. V(III) complexes with bidentate *N*,*N*-chelating iminopyrrolyl ligands also demonstrated stability in the base ligand environment and relatively high catalytic activity in the polymerization of ethylene when using Et_2_AlCl as an activator; in the presence of Et_3_Al or MAO, PE was formed in trace amounts [205]. The complexes with tridentate iminopyrrolyl and tetradentate bis(iminopyrrolyl) ligands [206], well as with bidentate phenoxy-phosphine ligands [207] and with tridentate 2,6-bis(diphenylhydroxymethyl)pyridyl ligand [208], have demonstrated similar behavior. However, in the last case, when comparing V(III) and V(V) derivatives in Et_2_AlCl-activated polymerization, the V(V) complex demonstrated higher activity. Apparently, even when using the same Cl-containing activator, the nature of the active site essentially depends on the nature of the polydentate ligand.

**Figure 25:**
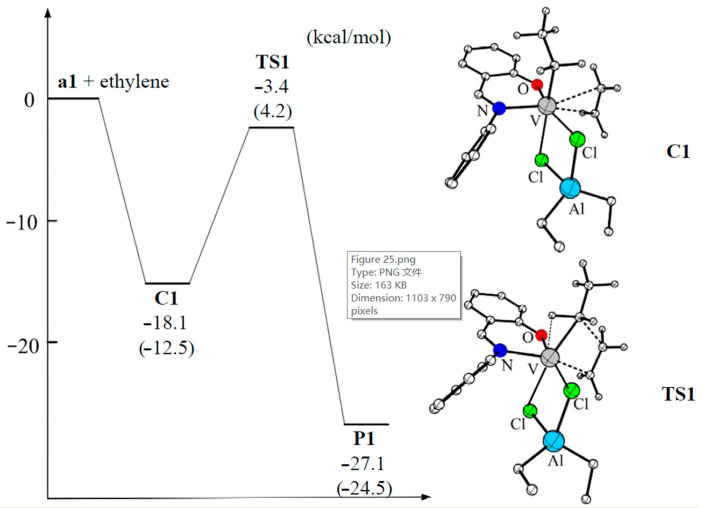
Potential energy surface and optimized geometries of the π-complex and transition state of the ethylene insertion catalyzed by the active species **V04**. The energy was calculated at the M06-L functional level, with energy changes calculated at the B3LYP-D3 functional level given in parentheses. Reprinted with permission from [211]. Copyright (2015) Elsevier B.V.

V(III) complexes bearing salicylaldiminato ligands (similar to **V04**), synthesized by Lee et al. [209,210], also showed high activity when using Et_2_AlCl as an activator. In their later work [211], these same authors reported the results of theoretical studies of the mechanism of ethylene polymerization with **V04**-type complexes. Actually, **V04** was chosen for DFT calculations, together with the consideration of Et_2_AlCl-free species, in the modeling of ethylene insertion in the framework of the Cossee–Arlman mechanism (Figure 25). In the first step, the ethylene molecule coordinates to the vacant site in **V04**, forming a π-complex (–18.1 kcal·mol^−1^). Subsequently, ethylene inserts into the vanadium–carbon bond via the four-membered cyclic transition state which has an activation energy barrier of 14.7 kcal·mol^−1^ relative to the π-complex. The overall ethylene insertion was found to be highly exothermic (–27.1 kcal/mol), and insertion product resembled the starting structure of **V04**. For hypothetical cationic active species [(PhN=CHC_6_H_4_O)VEt(THF)]^+^, ethylene complexation was less exothermic and the insertion barrier was higher by 5.7 kcal/mol. An additional argument in favor of the V–(μ-Cl)_2_–Al model was a clear correlation between the results of modeling and polymerization experiments for V(III) complexes with substituted salicylaldiminato ligands.

**Scheme 30:**
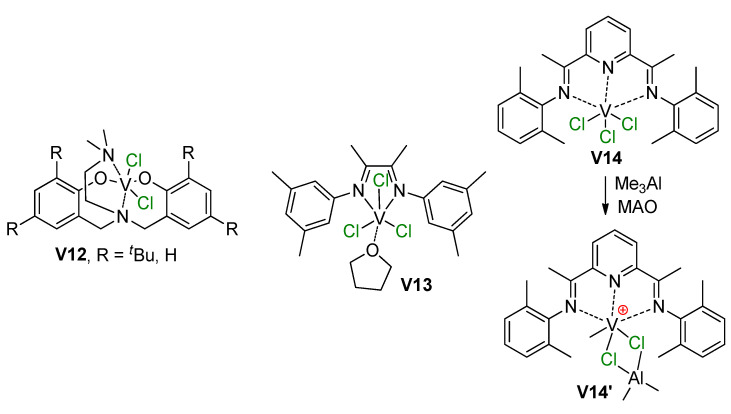
V(III) pre-catalysts **V12**–**V14** studied recently [212,213] and the formation of V–(μ-Cl)–Al cationic active species, confirmed by NMR experiments [213].

The results of relatively recent studies on V-catalyzed polymerization also confirm a distinct ‘chlorine effect’. In this regard, the study by Białek and Bisz [212] deserves a separate mention. In the polymerization of ethylene, ONNO-type bis(phenolates) **V12** (Scheme 30) were manyfold more active in the presence of Et_2_AlCl in comparison with MAO and perfluoroaryl borates. In ethylene/oct-1-ene copolymerization, the difference in activities decreased when using Et_2_AlCl and ^3^Bu_3_Al/[Ph_3_C][B(C_6_F_5_)_4_], but higher comonomer incorporation was observed in the first case.

In 2018 [213], Talsi et al. reported the results of the study of α-diimine (**V13**) and bis(imino)pyridine (**V14**) trichloro complexes of V(III) (Scheme 30) with the use of MAO, Me_2_AlCl, Me_2_AlCl/[Ph_3_C][B(C_6_F_5_)_4_], and Me_3_Al/[Ph_3_C][B(C_6_F_5_)_4_] activators. Through careful NMR experimentation, they demonstrated the formation of **V13**-based cationic complexes without V–(μ-Cl)–Al bonding, whereas **V14** formed V(Me)–(μ-Cl)_2_–Al species **V14′**. Notably, when Me_2_AlCl alone was used, an inactive V(Cl)–(μ-Cl)_2_–Al complex was the main reaction product.

In a series of recent publications [214,215,216], Nomura et al. presented the results of the study of the activation of V complexes with the use of V K-edge X-ray absorption near-edge structure analysis (XANES) and extended X-ray absorption fine structure (EXAFS) analysis. Regarding V(V) pre-catalysts of (imido)VCl_2_(OAr), (imido)VCl_3_, and similar types, as well as VOCl_3_, these studies clearly indicated the presence of V(III) species in the reaction mixture after activation by the organoaluminum compounds Me_2_AlCl, Et_2_AlCl, and EtAlCl_2_. In this way, the attribution of **V03** (Scheme 26) to active species should be corrected. However, no significant changes in either the oxidation state or the basic geometry were observed when (imido)vanadium(V) complexes were treated with MAO. In this way, the role of R_2_AlCl is in both the formation and stabilization of catalytic species.

**Figure 26:**
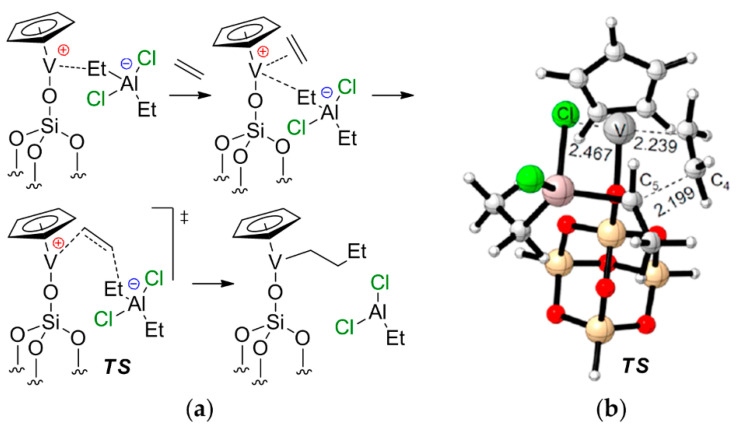
(**a**) Proposed mechanism of ethylene coordination/insertion on the supported CpV–Et_2_AlCl complex; (**b**) optimized geometry of the key transition state. Reprinted with permission from [217]. Copyright (2020) Elsevier B.V.

In conclusion, it should be noted that the chemistry of V-based single-site catalysts of α-olefin and diene polymerization primarily focuses on post-metallocene-type complexes. V-based catalysts exhibit some similarities to Ti(III) Ziegler–Natta catalysts (see **V07** in Scheme 27); however, half-sandwich and sandwich complexes of V have not found wide application. What is more interesting is the possible role of V–(μ-Cl)–Al bonding in the catalytic behavior of Cp-ligated complexes of vanadium. Recent qualitative research of the catalytic activity of *supported* Cp_2_V, carried out by Liu et al. [217], sheds some light on the issue. The authors suggested the formation of cationic CpV–O–Si(μ-O)_3_(silica) species under the action of Et_2_AlCl, and proposed a non-trivial mechanism of ethylene coordination/insertion with a marked V–Al cooperative effect (Figure 26). The stages of chain initiation and release were also studied. The activation barrier of the chain propagation was found to be ~16 kcal·mol^−1^.

## 5. Complexes of Cr

### 5.1. Selective Oligomerization of Ethylene Catalyzed by Cr Complexes

**Scheme 31:**
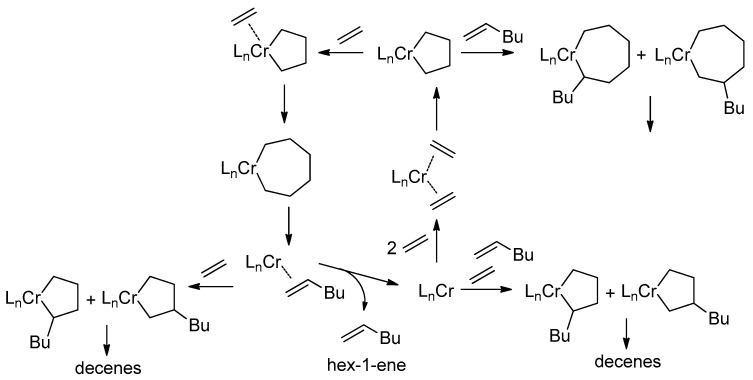
The main idea of metallacyclic mechanism of selective trimerization of ethylene, catalyzed by Cr complexes [219].

The selective oligomerization of ethylene with the use of Cr catalysts, resulting in the formation of hex-1-ene and oct-1-ene, is becoming an important contemporary industrial process [14]. The mechanism of the selective trimerization of ethylene [13,47,48,218] qualitatively differs from the Cossee–Arlman mechanism of polymerization and non-selective oligomerization of α-olefins by the coordination of two olefin molecules and the intermediate formation of metallacyclic species. As a result, when using selective trimerization catalysts, isomeric decenes are formed [219,220] (Scheme 31). The reaction intermediates and products, presented in this scheme, only provide a general idea of the Cr-catalyzed trimerization. Cr catalysts, unlike half-sandwich Ti complexes (Scheme 10), are of a more complex nature, selective tetramerization is possible, and the oxidation numbers of Cr reaction intermediates still are subject of discussion.

The Cr-catalyzed selective tri- and tetramerization of ethylene has been the subject of numerous reviews which have discussed both the practical and theoretical aspects of the reaction [13,14,47,48,84,221,222,223,224,225,226,227]. Pyrrole-based Cr catalysts were historically the first system for the synthesis of hex-1-ene [228], which is widely used in the petrochemical industry. Other groups of homogeneous Cr catalysts of tri-/tetramerization often outperform Cr/pyrrole systems on catalytic activity, but research is still ongoing.

At the very beginning of the study of Cr oligomerization catalysts, a substantial ‘chlorine effect’ was detected: Cr/pyrrole systems only demonstrated high activity in the presence of Et_2_AlCl/Et_3_Al mixtures of the activators. This fact was explained successfully from a mechanistic point of view (see Section 5.2 below), and the possible importance of Cr–(μ-Cl)–Al bonding in Cr-based catalytic species was not completely ignored during the ligand-oriented design of new single-site oligomerization catalysts, accompanied by oligomerization experiments.

### 5.2. Cr Complexes with Pyrrole and Similar Ligands

**Scheme 32:**
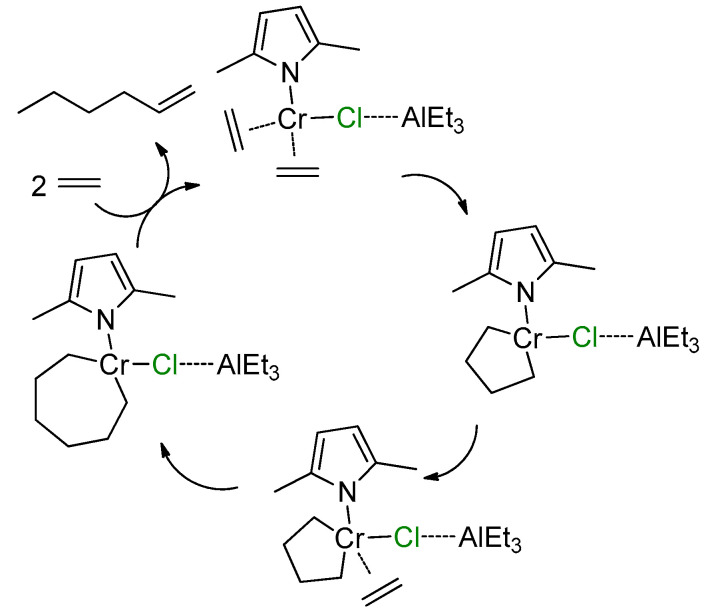
Proposed metallacycle mechanism for the Cr/pyrrolyl-catalyzed trimerization of ethylene involving Cr–(μ-Cl)–Al species [234].

Discovered in the late 1980s by Reagan [228], catalytic systems containing Cr(III) 2-ethylhexanoate, 2,5-dimethyl-1*H*-pyrrole, Et_2_AlCl, and Et_3_Al were optimized to a 1:3:8:11 ratio during the further research by Phillips Petroleum and Mitsubishi [229,230,231,232,233]. This catalyst is commonly known as the Chevron–Phillips ethylene trimerization system. In augmenting the main idea of the metallacyclic mechanism, the scientists of Sasol Technology proposed a novel mechanistic concept, which included the formation of Cr–(μ-Cl)–Al catalytic species [234]. They suggested that the Cr(II) center coordinates two ethylene molecules. Then, a Cr(IV) metallacycle is formed, subsequent π-coordination/insertion of the ethylene molecule results in a seven-membered metallacycle which is further subjected to reductive eliminative intramolecular β-hydrogen migration to the ζ-carbon followed by the coordination of two ethylene molecules with hex-1-ene release (Scheme 32).

During DFT modeling, unsubstituted pyrrole was used as a ligand, with the consideration of possible η^5^- and κ^1^-coordination. In addition, optimizations were performed for catalytic species with and without Me_3_Al coordination (‘Cl’ and ‘Me_3_AlCl’ models, respectively). Based on the results of the modeling, the triplet spin states for both Cr(II) and Cr(IV) were predicted to be the ground state. When comparing Cl and Me_3_AlCl models, significant lowering of the activation energy of the rate-limiting step by 11.3 kcal·mol^−1^ was found for the catalytic species with Cr–(μ-Cl)–Al bonding.

**Scheme 33:**
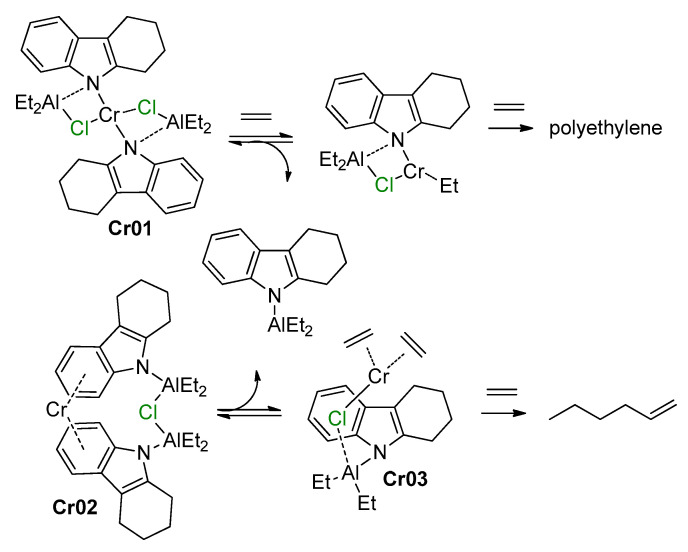
Structural formula and catalytic activity of 2,3,4,5-tetrahydro-1*H*-carbazole complexes **Cr01** and **Cr02** [235].

**Figure 27:**
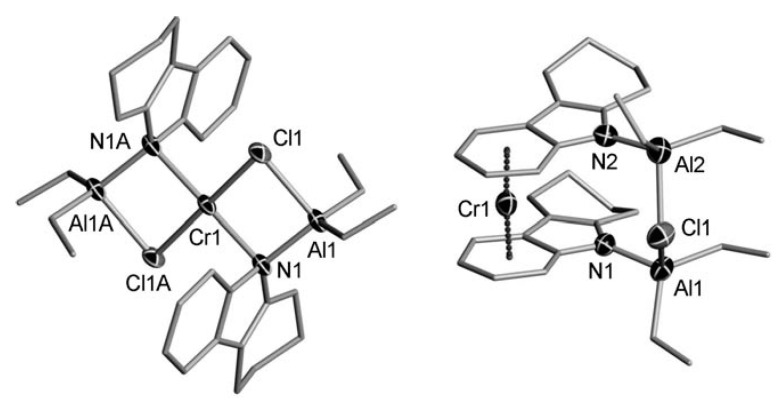
Molecular structures of the complexes **Cr01** and **Cr02**. Reprinted with permission from [235]. Copyright (2008) Wiley-VCH Verlag GmbH & Co.

In follow-up studies of pyrrole-based catalytic systems, Gambarotta, Duchateau et al., after failed attempts to isolate single crystals of 2,5-dimethyl-1*H*-pyrrole derivatives, obtained characterizable complexes of 2,3,4,5-tetrahydro-1*H*-carbazole [235]. In particular, by the treatment of [CrCl_3_(THF)_3_] or [CrCl_2_(THF)_2_] with a mixture of the ligand and Et_3_Al, the square-planar Cr(II) complex **Cr01** was obtained as a blue paramagnetic crystals. When chromium(III) 2-ethylhexanoate was used, the presence of AlEt_2_Cl was crucial, and the reaction afforded the new paramagnetic Cr(I) complex **Cr02**. Complex **Cr02** was also obtained by the reduction of **Cr01** using potassium (Scheme 33). The molecular structures of **Cr01** and **Cr02** are presented in Figure 27.

Complex **Cr01** catalyzed the polymerization of ethylene. In methylyclohexane, complex **Cr02** was an unprecedented single-component trimerization catalyst, producing hex-1-ene with only trace amounts of higher oligomers. The structure of the possible active complex **Cr03** is also presented in Scheme 33. The role of Cr–(μ-Cl)–Al bonds in the formation of the ligand environment of the catalytic center in **Cr03** seems clear.

**Scheme 34:**
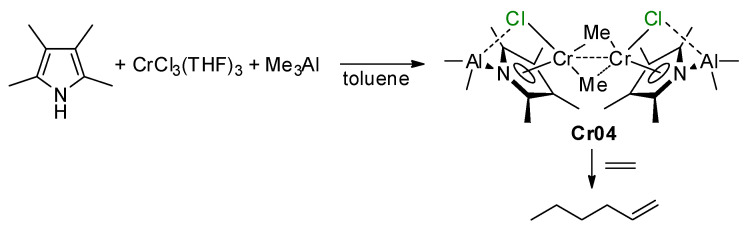
Synthesis and catalytic behavior of complex **Cr04** [236].

**Figure 28:**
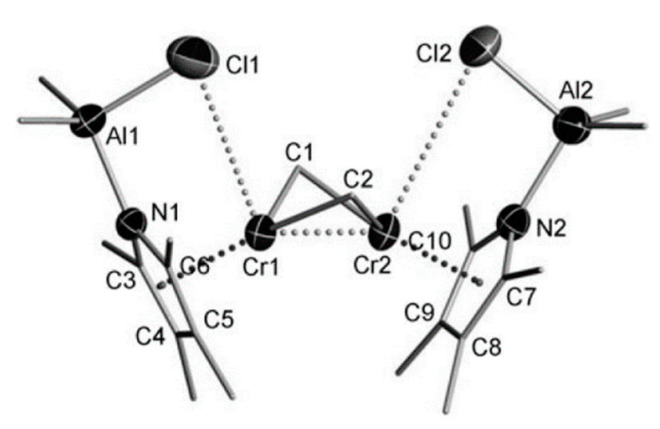
Molecular structures of complex **Cr04**. Reprinted with permission from [236]. Copyright (2009) Wiley-VCH Verlag GmbH & Co.

One year later, the same research team reported the synthesis of the 2,3,4,5-tetramethyl-1*H*-pyrrole-based Cr–(μ-Cl)–Al complex **Cr04** [236] (Scheme 34). Similarly to complex **Cr02**, in methylyclohexane, complex **Cr04** turned out to be the highly active ethylene trimerization catalyst without any activators. Apparently, the dimer of **Cr04** (Figure 28) dissociates in the solution with the retention of Cr–(μ-Cl)–Al coordination in the monomeric complex with an easily recognizable ‘constrained geometry’ structural motif.

Taking into account the results of theoretical [234] and experimental [235,236] studies of Cr/pyrrole systems, Budzelaar offered his own mechanistic concept, based on the DFT modeling of Cr/1*H*-indole catalytic systems [237]. The modeling results indicated that, in addition to the Cr(II)/Cr(IV) cycle (Scheme 33), Cr(I)/Cr(III) cycle, based on [(Indol-1-yl)⋯AlMe_2_(μ-Cl)Cr] species (**Cr05**), the role of the Cl atom was to stabilize metallacyclic intermediates and to block additional coordination of the ethylene molecule at the stage of hex-1-ene formation.

**Figure 29:**
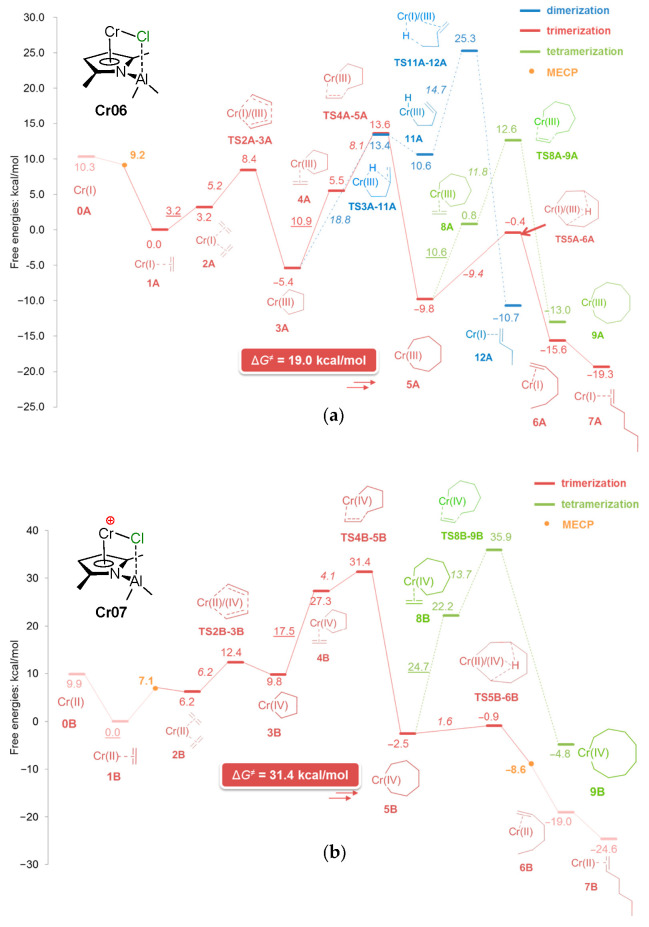
Calculated free energy diagram for model catalytic species **Cr06** (**a**) and **Cr07** (**b**). Me_2_Al fragments are omitted. Reprinted with permission from [238]. Copyright (2014) American Chemical Society.

Finally, to determine the problem of the oxidation states of the Cr catalytic center in the Chevron–Phillips ethylene trimerization system, Liu et al. carried out a detailed theoretical study of the model catalyst species **Cr06** and **Cr07** (Figure 29) based on 2,5-dimethyl-1*H*-pyrrole [238]. Optimizations showed that the retention of Cr–(μ-Cl)–Al bonding (strong or weak, d(Cr–Cl)) was changed in the interval of 2.5–4.0Å in all key reaction intermediates and transition states. Calculated free energy profiles (Figure 29) clearly showed the preference of the choice of **Cr06** as a model catalytic species—free activation energies were 19.0 and 31.4 kcal·mol^−1^ for **Cr06** and **Cr07**-based reaction pathways, respectively. In this way, the mechanistic concept of Cr(I)/Cr(III) catalytic cycle received additional confirmation.

Recently, the activation of the Chevron–Phillips ethylene trimerization system was studied by Tromp et al. using catalytic and spectroscopic (XAS, EPR, UV–vis) experiments under industrial conditions [239]. It was found that 2,5-dimethyl-1*H*-pyrrole reacts with Et_2_AlCl и Et_3_Al with the formation of [2,5-dimethyl-2*H*-pyrrole]⋯AlEt_3_ and [μ-2,5-dimethyl-1*H*-pyrrole](μ-Cl)(AlEt_2_)_2_ complexes, and their reaction with Cr(III) 2-ethylhexanoate results in polymeric Cr(II) pyrrolyl species containing Et_2_AlCl fragments. However, the structure of real catalytic species remains unknown.

### 5.3. Cr Aminodiphosphine Complexes

Among others, Cr(III) derivatives of chelating ligands of the formula R^1^R^2^P–N(R^3^)–PR^4^R^5^ (PNP–Cr complexes) represent pre-catalysts of the selective tri-/tetramerization of ethylene which are characterized by high catalytic activity and stability over time [14,47,84,221,222,223,224,225,240]. Usually, the activation of PNP–Cr pre-catalysts is performed by the reaction of L^PNP^CrCl_3_ complexes (L^PNP^—PNP ligand) or mixtures of L^PNP^ with Cr(III) salts by MAO. The use of R_3_Al/perfluoroaryl borate systems is rarely used [241] despite its undoubted merits. Evidently, the presence of R_2_AlCl species, arising as a result of the reaction between L^PNP^ZrCl_3_ and organoaluminum activators, implies the possible formation of Cr–(μ-Cl)–Al species. The results of studies of PNP–Cr catalysts in view of this possibility are summarized and discussed below in chronological order.

**Scheme 35:**
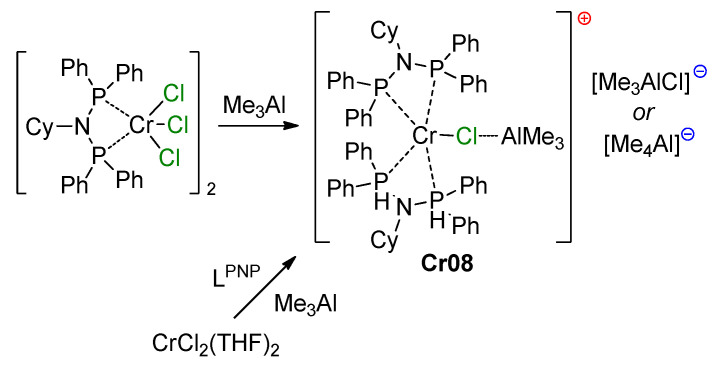
Formation of cationic complex **Cr08** [242].

**Figure 30:**
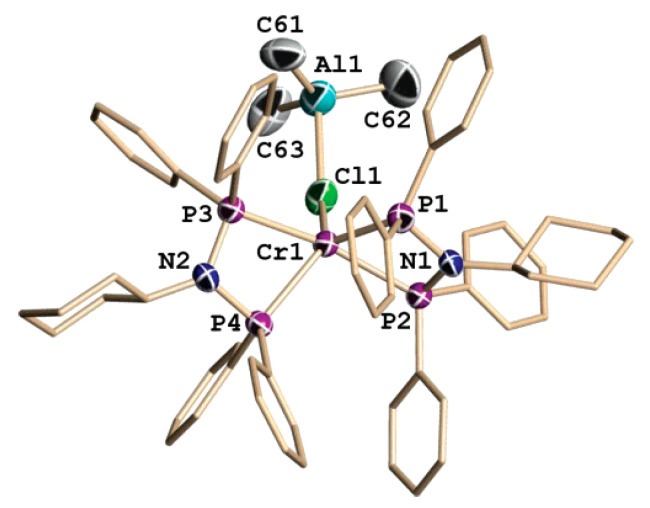
Plot of the Cr-containing cationic moiety of **Cr08** with thermal ellipsoids of the non-carbon atoms drawn at the 30% probability level. Reprinted with permission from [242]. Copyright (2006) American Chemical Society.

When studying the activation of [CyN(PPh_2_)_2_]CrCl_3_ by Me_3_Al in toluene (Cy—cyclohexyl, Scheme 35), Gambarotta, Duchateau et al. separated and characterized a cationic complex of Cr(II), **Cr08** [242]. L^PNP^ does not react with CrCl_2_(THF)_2_; nevertheless, in the presence of excess of Me_3_Al, **Cr08** was obtained in a high yield. XRD studies confirmed the molecular structure of **Cr08** (Figure 30). Being activated by MAO, this complex was highly active in the oligomerization of ethylene with the formation of oct-1-ene as the main reaction product.

The authors also proposed that the catalytic behavior of **Cr08** and the failure of L^PNP^ to ligate to a CrCl_2_ moiety in the absence of Me_3_Al suggest that the acquisition of a second ligand and cationization are central to the stabilization of a Cr(II) catalyst precursor. Considering that excess MAO is necessary for the activation, they also speculated that the dicationic [L^PNP^_2_Cr]^2+^ complex may be the actual catalytically active species. However, other hypotheses, involving the possibility of further abstraction of the [PNP] ligand from the ‘dormant’ catalyst precursor **Cr08**, cannot be ruled out [242]. For our part, we will merely add that in the latter case, the Cr(II) cation becomes open for both the π-coordination of ethylene and for Cr–(μ-Cl)–Al bonding with organoaluminum components of the catalytic system.

**Scheme 36:**
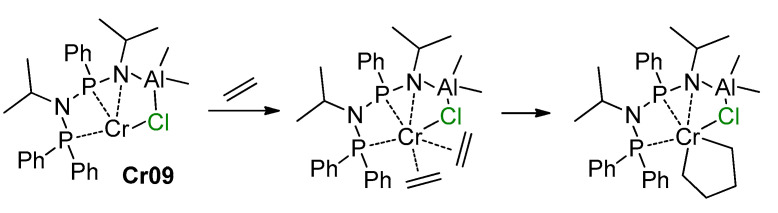
Supposed structure of the active PNPN-Cr catalyst **Cr09** [244]. Copyright (20XX) Wiley-VCH Verlag GmbH & Co. Copyright (2016) Royal Society of Chemistry. Copyright (2012) American Chemical Society. Copyright (2015) Springer Nature. Copyright (2019) Elsevier B.V.

PNP–Cr complexes containing diphosphine ligands with two amine groups at one of the phosphorus atoms (L^PNPN^) are of interest to researchers due to the ability of similar pre-catalysts to be activated by Et_3_Al, without the use of MAO [243]. Evidently, such unconventional reactivity forces the assumption that L^PNPN^ can form catalytic species which differ from L^PNP^-based species. In [244], Müller et al. reported a study of the interaction of Et_3_Al and Me_3_Al with the Ph_2_PN(^i^Pr)P(Ph)NH^i^Pr ligand. During this reaction, rearrangement to the [^i^PrNP(Ph)P(Ph)_2_=N^i^Pr ligand occurred. The complex of this ligand with CrCl_3_ was inactive in oligomerization. After preparation of the active model pre-catalyst [Ph_2_PN(^i^Pr)P(Ph)N^i^Pr]Cr(Cp)Cl, it was concluded that the active catalytic species **Cr09** exhibits a binuclear nature (Scheme 36). During further research [245], it was shown that the PNPN–Cr system, generated by the reaction of Cr(III) acetylacetonate (Cr(acac)_3_) with L^PNP^ and Et_3_Al, is inactive in oligomerization. At the same time, the addition of Cl-containing compounds of various nature resulted in the selective trimerization of ethylene.

**Scheme 37:**
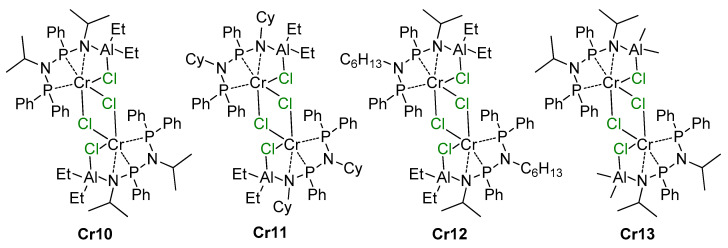
Cr_2_Al_2_ complexes **Cr10**–**Cr13** [246].

**Figure 31:**
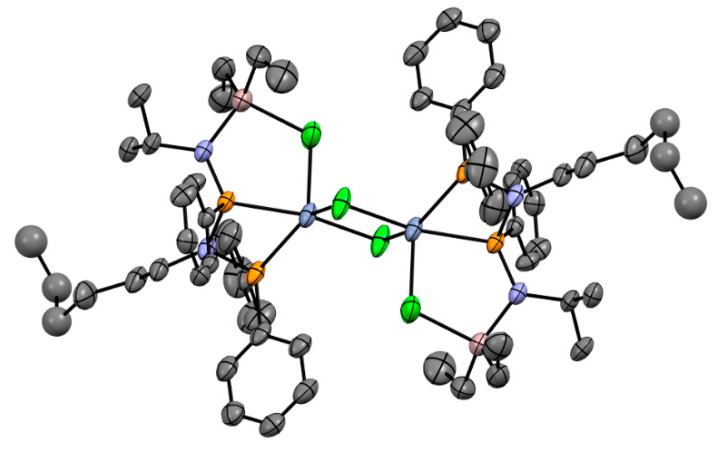
Molecular structure of complex **Cr12** [246].

Attempts to isolate PNPN-based Cr–Al species have been successful; Cr_2_Al_2_ complexes **Cr10**–**Cr13** (Scheme 37) were obtained by the reaction of L^PNPN^CrCl_3_ with Et_3_Al, or by the interaction of Al derivatives of L^PNPN^ with CrCl_2_(THF)_2_ [246]. Molecular structures of all complexes were determined by XRD (see Figure 31 for an example). It was found that **Cr10** is inactive in ethylene oligomerization, indicating that **Cr10** is not a self-activating complex and that the oxidation state of Cr(II) is not sufficiently low to form hex-1-ene. Hence, the formation of **Cr09**-type catalytic species needs dissociation and further reduction.

However, the studies listed above only contain suggestions about the nature of catalytic species in the systems containing PNP–Cr complexes and R_2_AlCl. In 2016, Evans et al. reported the results of a study of [^i^PrN(PPh_2_)_2_]CrCl_3_ activation by Me_3_Al [247]. The Cr K-edge XAFS spectrum after 1 min indicated the formation of [^i^PrN(PPh_2_)_2_]CrClMe(μ-Cl)AlMe_3_(THF) and then [^i^PrN(PPh_2_)_2_]CrMe(μ-Cl)AlMe_3_. Such species are evidently able to form Cr(II) cations under the action of MAO; however, given that PNP–Cr catalysts usually operate under relatively low Al_MAO_/Cr ratios (~100), in our view, the possibility of the intermediate coordination of R_2_AlCl at the Cr center seems possible.

**Scheme 38:**
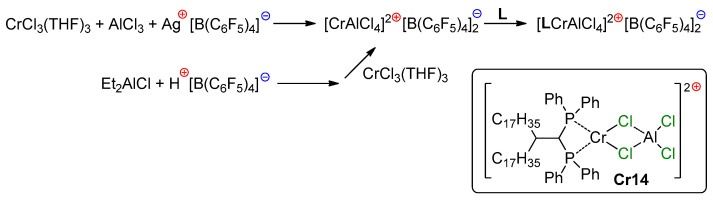
Formation of dicationic complex **Cr14** [241].

When studying the use of perfluoroaryl borates for the activation of PNP–Cr pre-catalysts, Lee et al. proposed an original method for the synthesis of Zr–Al dicationic complex **Cr14** [241] (Scheme 38). This complex was found to be highly active in the selective tetramerization of ethylene, but a fairly large amount of PE (1.7 wt.%) was concomitantly generated. Further ligand design (introduction of SiR_3_ substituents in *p*-positions of the Ph rings in L^PNP^) resulted in an increase in the activity and suppression of the PE formation.

Summarizing the above-mentioned research, in view of the huge number of the articles on the PNP–Cr-catalyzed tri/tetramerization of ethylene, the role of R_2_AlCl⋯Cr bonding was not examined in most studies. In many cases, this was due to the methodology of the experiment on pre-catalyst preparation and activation, based on the mixing of a soluble Cr source (for example, Cr(acac)_3_) and L^PNP^, followed by treatment with MAO. This, among other things, may be due to the low solubility and varied composition of the commonly used starting complex CrCl_3_(THF)_3_, which complicates the separation and purification of L^PNP^CrCl_3_ pre-catalysts (for instance, we have been unable to solve another similar problem, presented in [248]). The complex [CrCl_2_(μ-Cl)(THF)_2_]_2_, recently synthesized by Lee et al. [249], seems to be a more convenient and reliable starting compound for the synthesis of L^PNP^CrCl_3_. We can assume that more studies on the effect of the use of [CrCl_2_(μ-Cl)(THF)_2_]_2_, or additional amounts of R_2_AlCl, on catalytic activity of known ‘chlorine-free’ PNP–Cr systems, may lead to further improvements in the PNP-based catalysts of ethylene tri- and tetramerization.

### 5.4. Other Cr Complexes in the Single-Site Catalysis of Oligomerization and Polymerization

**Figure 32:**
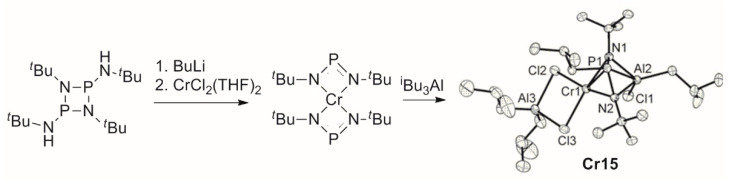
Preparation and molecular structure of complex **Cr15**. Reprinted with permission from [250]. Copyright (2008) Wiley-VCH Verlag GmbH & Co.

The range of chelating ligands for the synthesis of Cr-based oligomerization and polymerization of single-site catalysts is not limited by PNP-type compounds. Thus, for example, complex **Cr15** was synthesized by the reaction of the *^t^*BuNPN*^t^*Bu dianion with Cr chlorides, followed by treatment with ^i^Bu_3_Al [250] (Figure 32). This complex was found to be a single-component catalyst of ethylene polymerization.

**Figure 33:**
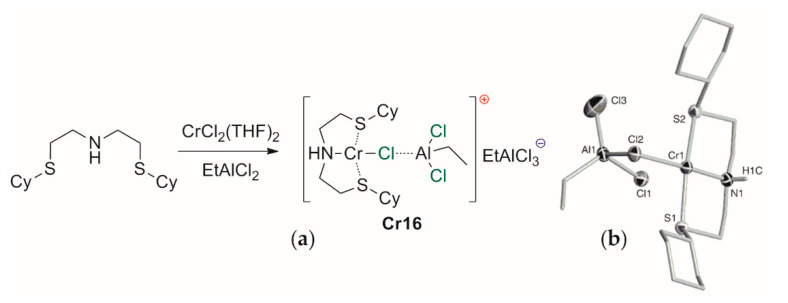
(**a**) Synthesis of complex **Cr16**; (**b**) ORTEP view of **Cr16** (thermal ellipsoids set at 30%), with the Al-containing counterion omitted for clarity. Reprinted with permission from [252]. Copyright (2006) Wiley-VCH Verlag GmbH & Co.

HN(CH_2_CH_2_PPh_2_)- and HN(CH_2_CH_2_SR)_2_-based Cr(II) and Cr(III) chloro complexes were synthesized by McGuinness et al. [251]: the treatment of [HN(CH_2_CH_2_PPh_2_)]CrCl_2_ by 1,4-diazabicyclo[2.2.2]octane (DABCO) resulted in dimer {[N(CH_2_CH_2_PPh_2_)]Cr(μ-Cl)}_2_ and its –SR analogs. Further studies of the activation of these complexes by MAO and Et_3_Al/B(C_6_F_5_)_3_ indicated that the Cr(II)/Cr(IV) cycle is preferable for this type of catalyst. When HN(CH_2_CH_2_SCy)_2_ was treated with CrCl_2_(THF)_2_ and EtAlCl_2_, complex **Cr16** was obtained (Figure 33) [252]. This Cr(II) complex, after activation by MAO, was inferior to SNS Cr(III) complexes in catalytic activity in the trimerization of ethylene.

**Figure 34:**
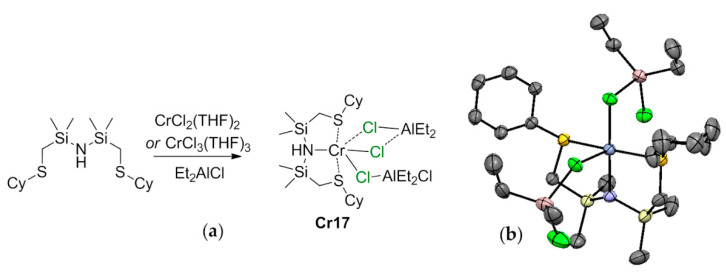
(**a**) Synthesis of complex **Cr17**; (**b**) ORTEP view of **Cr17** (hydrogen atoms are omitted) [253]. Copyright (20XX) Wiley-VCH Verlag GmbH & Co. Copyright (2016) Royal Society of Chemistry. Copyright (2012) American Chemical Society. Copyright (2015) Springer Nature. Copyright (2019) Elsevier B.V.

**Figure 35:**
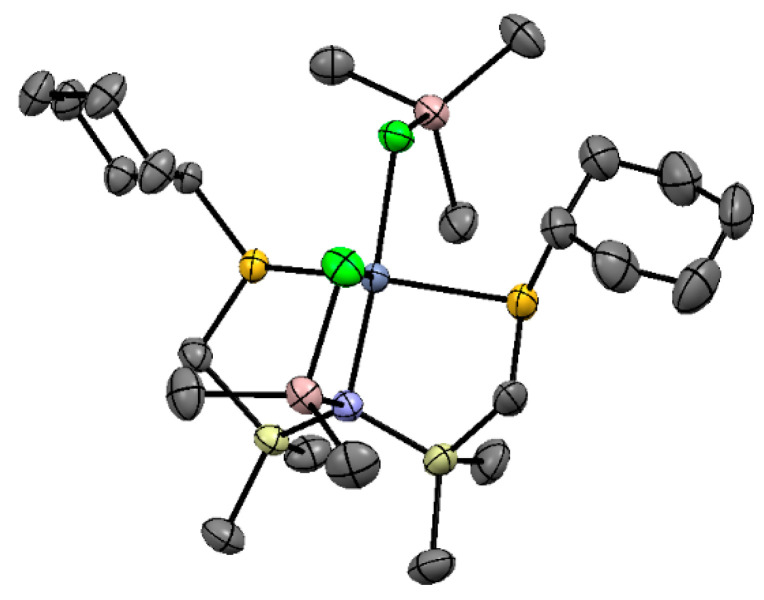
Molecular structure of complex **Cr18** [253].

In 2011, the SNS ligand was modified by replacing CH_2_ fragments near the N atom with SiMe_2_ groups (HN(CH_2_CH_2_SR)_2_, R = Cy, *^t^*Bu, Ph). The reaction of HN(CH_2_CH_2_SCy)_2_ with Et_2_AlCl and Cr(III) or Cr(II) chlorides afforded the Cr(II) complex **Cr17** (Figure 34) [253]. In the presence of MAO (500–1000 eq.), this complex catalyzed the oligomerization of ethylene (mainly trimerization). After abstraction of the N–H proton during the synthesis of the AlMe_2_ analog of **Cr17**, complex **Cr18** was obtained (Figure 35): its selectivity in the oligomerization of ethylene was even lower.

**Scheme 39:**
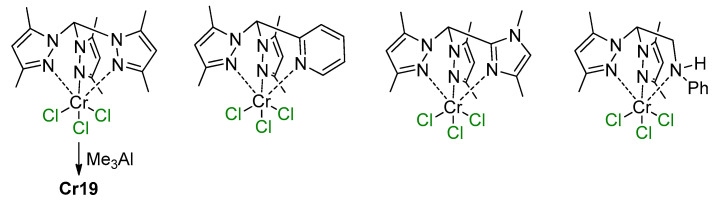
CrCl_3_ complexes with tris- and bis-pyrazolyl ligands [254].

**Figure 36:**
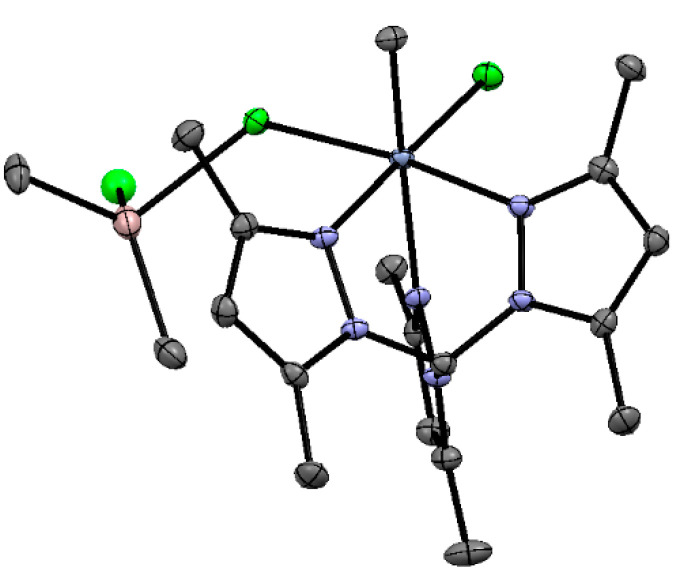
Molecular structure of complex **Cr19** [254].

A similar pattern was observed when studying Cr complexes with tris- and bis-pyrazolyl ligands [254] (Scheme 39). Depending on the absence or the presence of the –NH– fragment in the bridging ligand, the reaction of LCrCl_3_ with Me_3_Al resulted in the formation of Cr–(μ-Cl)–Al complexes with different types of Al–N bonding. After treatment with 200 eq. MAO, the activities of LCrCl_3_ and Cr–(μ-Cl)–Al complexes (for example, **Cr19**, Figure 36) in the oligomerization of ethylene were close, with the same oligomer distributions. In this way, under the action of MAO, organoaluminum chloride is eliminated finally and irreversibly.

**Figure 37:**
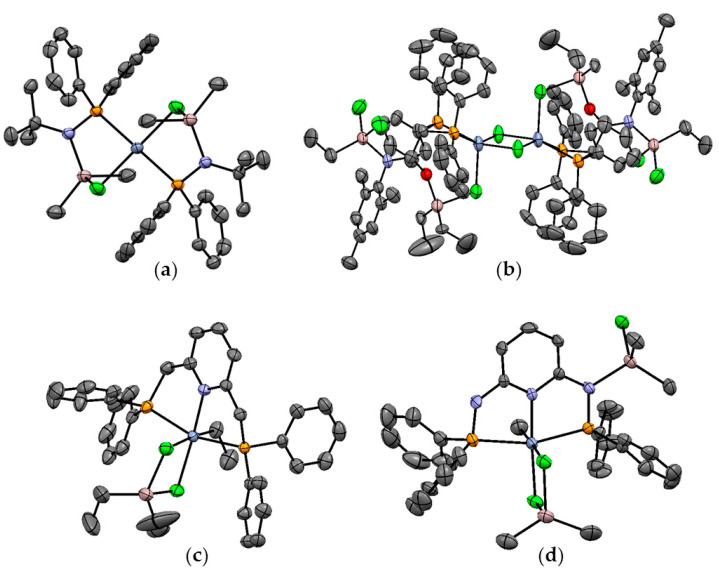
Examples of the molecular structures of the complexes containing Cr–(μ-Cl)–Al fragment (XRD analysis data), (**a**) **Cr20** [255]; (**b**) **Cr21** [256]; (**c**) **Cr22** [257]; (**d**) **Cr23** [257].

In the early 2010s, a number of articles were devoted to the preparation and catalytic studies of Cr complexes with bi- and polydentate ligands of different nature, including the synthesis and characterization of Cr–(μ-Cl)–Al complexes: in particular, Cr derivatives of Ph_2_PN*^t^*Bu [255], (Ph_2_P)_2_CHCO_2_^−^ and (Ph_2_P)_2_C=C(NHR)O^−^ [256], 2,6-bis(CH_2_PPh_2_)pyridine [257], 2,6-bis(NH=PPh_2_)pyridine [258], 2-(NHCH_2_PPh_2_)pyridine [259], and Ph_2_C(1*H*-pyrrol-2-yl) [260]. In several studies, an explicit ‘chlorine effect’ was detected, although most of them included XRD data for isolated Cr–(μ-Cl)–Al molecules (Figure 37). However, as opposed to the relatively well-studied Chevron–Phillips ethylene trimerization system and its analogs, we are not even close to understanding the role of Cr–(μ-Cl)–Al bonding in the catalytic chemistry of Cr-based post-metallocenes.

## 6. Complexes of Ni

Complexes of Ni are intensively being studied as efficient catalysts for the coordination oligomerization (SHOP process and beyond [46,261,262]) and polymerization [5,263,264,265] of α-olefins. Ni-based polymerization catalysts are very different from early transition metal (Ti, Zr, and V) complexes due to the lower sensitivity of Ni centers to electron-donor fragments, thus providing the copolymerization of α-olefins with polar vinyl monomers [266,267]. Another attractive property of Ni-based catalysts is their chain walking capability [268], which enables them to obtain branched polyolefins of different architectures. The direct participation of Ni–(μ-Cl)–Al species in catalytic processes at the Ni center is a matter of discussion; however, in the case of Ni, similar additional interactions are of dubious value. We have restricted the discussion in this section to a few examples of Ni–(μ-Cl)–Al bonding suspected of being associated with α-olefin polymerization.

**Figure 38:**
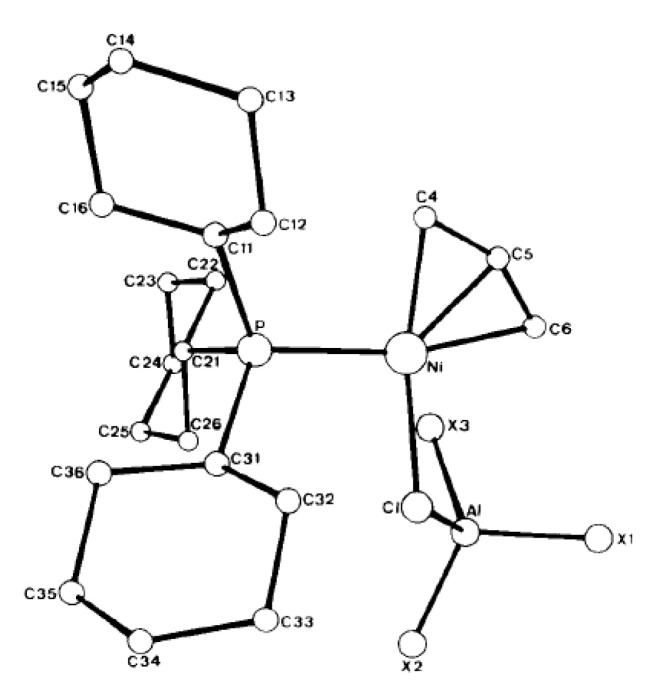
The molecular structure of complex **Ni01** comprising η^3^-allylnickel chloride, PCy_3_, and MeAlCl_2_ (X = Cl, Me). Reprinted with permission from [269]. Copyright (1979) Elsevier B.V.

The mere presence of Ni–(μ-Cl)–Al bonding in coordination compounds formed during the activation of Ni(II) chloro complexes by organoaluminum has been known for a fairly long time. Thus, for example, the formation of Cy_3_P(η^3^-allyl)Ni(μ-Cl)AlMeCl_2_ (**Ni01**) was clearly proven by XRD analysis [269] (Figure 38).

However, EXAFS studies of the reaction mixture formed from NiCl_2_(PEt_3_)_2_ and Me_3_Al_2_Cl_3_ [270] showed the presence of C and P atoms in the first coordination shell of Ni; more distant shells included Al (d = 3.0 Å) and Cl (d = 4.4 Å), thus excluding Ni–(μ-Cl)–Al bonding. Similar spectral studies were continued by de Souza et al. [271], for example, with MAO-activated Ni(α-diimine)Cl_2_. They showed that in the active catalyst species, the Ni(II) atom is surrounded by C and N atoms in the first shell and Cl atoms at a higher distance (~3.5 Å), and proposed that the Ni⋯Cl interaction plays an important, previously underestimated, role in the polymerization of olefins.

**Scheme 40:**
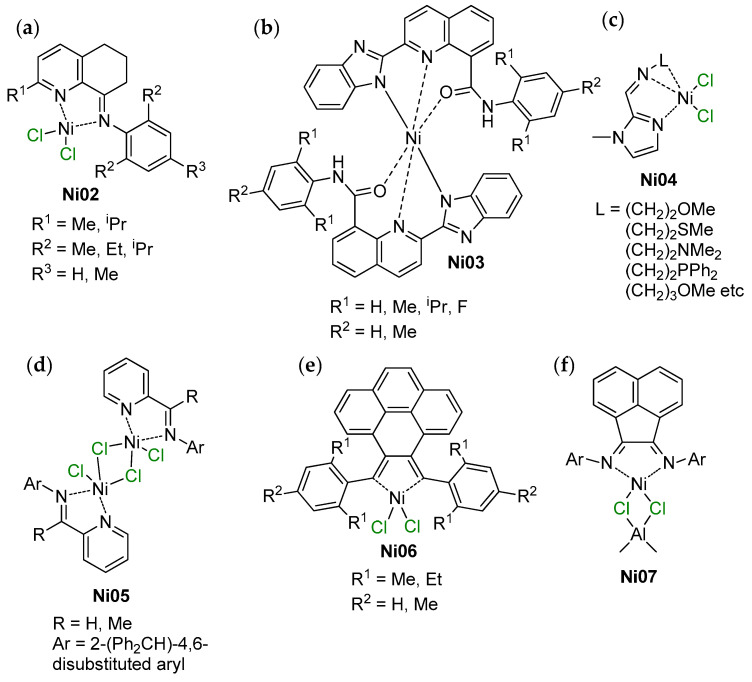
Structural formula of Ni pre-catalysts studied in α-olefin oligomerization and polymerization: (**a**) **Ni02 [272]**; (**b**) **Ni03** [273]; (**c**) **Ni04** [274]; (**d**) **Ni05** [275]; (**e**) **Ni06 [276]**; and (**f**) **Ni07** [277].

In a number of later studies, the catalytic activity of different complexes was studied with the use of R_x_AlCl_3–x_ activators. Thus, for example, a number of N-(5,6,7-trihydroquinolin-8-ylidene)arylamino complexes of Ni(II) (**Ni02** in Scheme 40) have demonstrated high activity in the oligomerization of ethylene with the formation of but-1-ene (100% C4, 79–98% α-C4 selectivity) [272]. Higher activities were achieved when using Me_3_Al_2_Cl_3_ instead of MAO. The authors have limited themselves to descriptions of the experimental results, without adding mechanistic interpretations. Complex **Ni03** with the 2-benzimidazolyl-N-arylquinoline-8-carboxamide ligand demonstrated lower activity and but-1-ene selectivity [273]; Et_2_AlCl was the best activator. (Iminoalkyl)imidazole complex **Ni04** showed similar behavior [274]. Et_2_AlCl and MAO equally successfully activated 2-iminopyridyl complex **Ni05** [275] and 4,5-bis(arylimino)pyrenylidene derivative **Ni06** [276] (Scheme 40).

As shown in [278], when using EtAlCl_2_ as an activator, ethylene oligomerization is complicated by Friedel–Crafts alkylation when using toluene as a reaction medium. Another side process with the participation of alkylaluminum chlorides is interaction with the polydentate ligand with the emergence of Ni coordination vacancies [279].

Recently, Soshnikov et al. have shown that α-diimine Ni(II) complexes under the action of R_2_AlCl form the Ni(I) complex **Ni07** with Ni–(μ-Cl)–Al fragments, representing the catalyst’s resting states [277]. It is quite possible that derivatives of similar species are essential to ensuring ‘living’ ethylene/α-olefin copolymerization with the formation of *block*-polyolefins [280].

## 7. Conclusions

In the present review, we have summarized and commented on the data pertaining to the participation of M–(μ-Cl)–Al bonding in the formation of transition metal complexes which are active in α-olefin (and diene) chemistry. Of particular interest were the polymerization and oligomerization of α-olefins. We hypothesize that the theory and practice of single-site polymerization and oligomerization, catalyzed by Ti, Zr, and Cr complexes, have suffered from the use of 10^2^–10^4^ equivalents of MAO for the activation of group 4 and 6 metal complexes in laboratory practice. In such conditions, consideration of the possibility of relatively weak M–(μ-Cl)–Al bonding makes no sense because MAO operates as an organoaluminum ‘sponge’. However, in recent years, with the growing interest in ‘low-MAO’ and ‘MAO-free’ catalytic processes, interest has re-focused on reversible M–(μ-Cl)–Al coordination on catalytic centers, as an additional factor affecting the chain propagation/chain termination balance, which appears to be reasonable.

The complex understanding of the nature of M–(μ-Cl)–Al bonds in metal complexes and reaction intermediates is significantly complicated by small amounts of fragmented data. For complexes with one bridging fragment, the (μ-Cl)–Al distance is not much different from the d(Al–Cl) value in R_2_AlCl (and the M–(μ-Cl) distance is lengthened in comparison with d(M–Cl) in mononuclear chloro complexes). However, when forming two bridging fragments, the values of d((μ-Cl)–Al) and d(M–(μ-Cl)) converge in magnitude. Apparently, the former M–(μ-Cl)–Al species can be considered as reactive intermediates, whereas the latter M–(μ-Cl)(μ-X)–Al species represent dormant sites that can be separated and characterized. Obviously, lower stability of the M–(μ-Cl)–Al species complicates their identification.

Additionally, in contrast to alkyl, H and F fragments that have been established in the formation of M–X–Al catalytic species, M–Cl–Al bonding in the reaction intermediates cannot be detected by the only convenient and reliable method of analysis of diamagnetic species, i.e., NMR spectroscopy. Other spectral methods leave space for interpretation; XRD analysis has limited use. In this context, it is reasonable that quantum-chemical modeling has been used extensively in the studies of M–Cl–Al-containing molecules and processes which utilize them. Additionally, during studies of the catalytic processes, the possibility of the formation of the M–(μ-Cl)–Al species should not be ignored, at least in order to separate and characterize metal complexes exhibiting a non-trivial structure or, in the end, to formulate more efficient catalytic systems.

## Figures and Tables

**Table 1:** Conversion and product yields in the reaction of the oct-1-ene with Me_3_Al in the presence of 2 mol% L_2_ZrCl_2_ (L_2_ZrCl_2_/alkene/Me_3_Al = 1:50:60, 20 °C, 24 h) [126].

L_2_ZrCl_2_	Solvent	Conversion, %	Product Yield, %			
			8	9	10	11
1a	CH_2_Cl_2_	92	3	14	7	68
	toluene	69	3	21	7	38
1b	CH_2_Cl_2_	84	11	14	7	52
	toluene	39	9	9	9	12
1c	CH_2_Cl_2_	68	53	8	7	-
	toluene	44	15	14	14	1
1d	CH_2_Cl_2_	24	<1	5	5	13
	toluene	13	<1	4	6	2
1i	CH_2_Cl_2_	89	31	19	-	39
	toluene	70	38	14	10	8
1j	CH_2_Cl_2_	0	-	-	-	-
	toluene	11	9	-	-	2
1k	CH_2_Cl_2_	2	0.6	0.3	0.7	
	toluene	4	<1	1.6	1	1
1l	CH_2_Cl_2_	30	20	1	<1	9
	toluene	99	63	2	-	34
1m	CH_2_Cl_2_	16	15	-	<1	<1
	toluene	26	22	-	1	3
1n	CH_2_Cl_2_	3	1	-	-	-
	toluene	35 ^1^	13	-	-	-
1o	CH_2_Cl_2_	17	16	<1	<1	-
	toluene	2	46	2	<1	4
Cp_2_ZrMe_2_	CH_2_Cl_2_	16	6	4	5	1
	toluene	9	3	3	<1	<1

^1^ Dimers and oligomers formed.

## Data Availability

Not applicable.
